# Focused CRISPR-Cas9 genetic screening reveals USO1 as a vulnerability in B-cell acute lymphoblastic leukemia

**DOI:** 10.1038/s41598-021-92448-w

**Published:** 2021-06-23

**Authors:** Amit Kumar Jaiswal, Hellen Truong, Tiffany M. Tran, Tasha L. Lin, David Casero, Michael O. Alberti, Dinesh S. Rao

**Affiliations:** 1 Department of Pathology and Laboratory Medicine, David Geffen School of Medicine At UCLA, Los Angeles, CA 90095 USA; 2 Molecular, Cellular and Integrative Physiology Graduate Program, UCLA, Los Angeles, USA; 3 Department of Internal Medicine, Division of Hematology/Oncology, UCLA, Los Angeles, USA; 4 F. Widjaja Foundation Inflammatory Bowel and Immunobiology Research Institute, Cedars Sinai Medical Center, Los Angeles, CA 90048 USA; 5 Department of Pathology, Washington University, St. Louis, USA; 6 Jonsson Comprehensive Cancer Center, UCLA, Los Angeles, USA; 7 Broad Stem Cell Research Center, UCLA, Los Angeles, USA

**Keywords:** Leukaemia, Cancer, Cancer genetics

## Abstract

Post-transcriptional gene regulation, including that by RNA binding proteins (RBPs), has recently been described as an important mechanism in cancer. We had previously identified a set of RBPs that were highly dysregulated in B-cell acute lymphoblastic leukemia (B-ALL) with *MLL* translocations, which carry a poor prognosis. Here, we sought to functionally characterize these dysregulated RBP genes by performing a focused CRISPR dropout screen in B-ALL cell lines, finding dependencies on several genes including *EIF3E*, *EPRS* and *USO1*. Validating our findings, CRISPR/Cas9-mediated disruption of *USO1* in *MLL-*translocated B-ALL cells reduced cell growth, promoted cell death, and altered the cell cycle. Transcriptomic analysis of *USO1*-deficient cells revealed alterations in pathways related to mTOR signaling, RNA metabolism, and targets of MYC. In addition, USO1-regulated genes from these experimental samples were significantly and concordantly correlated with *USO1* expression in primary samples collected from B-ALL patients. Lastly, we found that loss of *Uso1* inhibited colony formation of *MLL*-transformed in primary bone marrow cells from *Cas9-EGFP* mice. Together, our findings demonstrate an approach to performing focused sub-genomic CRISPR screens and highlight a putative RBP vulnerability in *MLL*-translocated B-ALL, thus identifying potential therapeutic targets in this disease.

## Introduction

B-ALL is the most common type of leukemia in the pediatric population, and is characterized by a number of recurrent chromosomal rearrangements^1–4^. Among these, the t (4;11) *MLL-AF4* (*KMT2A-AFF1*) translocation gives rise to a highly aggressive form of B-ALL^5,6^. Patients with MLL-rearranged B-ALL have a dismal prognosis, with 5-year event-free survival rates hovering at 33.6% for infants^7^ and 50% for older children and adults^8^. Most of these patients are resistant to conventional treatment with chemotherapy and steroids^9^, with bone marrow transplantation being the only curative therapeutic alternative^10^. Although recent developments such as CAR-T therapy^11^ and anti-CD19 based therapy such as Blinatumomab^12^ have raised hope for such patients^13^, antigen escape and lineage infidelity in *MLL*-translocated leukemia have proved problematic^14^. Therefore, there is an urgent need to better characterize potential therapeutic targets with high specificity.

The *MLL-AF4* translocation engenders a unique transcriptional profile, as the fusion protein juxtaposes a histone methyltransferase (MLL, also known as KMT2A) with a protein that is involved in transcriptional regulation (AF4, or AFF1). Recently our lab carried out a study examining the expression of RBPs in B-ALL^15^, including both known and predicted RBPs^16^. In our analysis, we identified 36 RBPs that are highly upregulated in *MLL-AF4* translocated B-ALL^15^. To study the importance of these genes in B-ALL, we implemented the powerful gene editing technique, CRISRP/Cas9, to perform a rapid and medium-throughput assessment of gene function. Genome wide CRISPR/Cas9 screens on AML cell lines have identified multiple gene targets critical for cell proliferation and survival^17^, but similar studies have not been performed in *MLL-AF4* translocated B-ALL.

In the present study, we performed a sub-genomic CRISPR/Cas9 dropout screen using 36 highly upregulated RBPs in primary human B-ALL and identified several novel vulnerabilities that included three putative RBPs. Of these, *USO1*, a putative RBP and a known regulator of vesicular transport, was identified as a *MLL-AF4* target gene. CRISPR/Cas9-mediated disruption of *USO1* significantly altered cell growth and the cell cycle in B-ALL cell lines; as well as inhibited the colony forming potential of *MLL*-transformed primary murine bone marrow cells. *USO1* depletion regulated the expression of genes related to mTOR signaling, metabolism of RNA, and MYC targets. Together, our studies provide a comprehensive rubric to functionally evaluate putative targets identified from expression profiling, and the identification of a novel potential target in *MLL*-rearranged leukemia.

## Results

### CRISPR/Cas9 screen identifies potential vulnerabilities in MLL-AF4 leukemic cell growth

**Figure 1:**
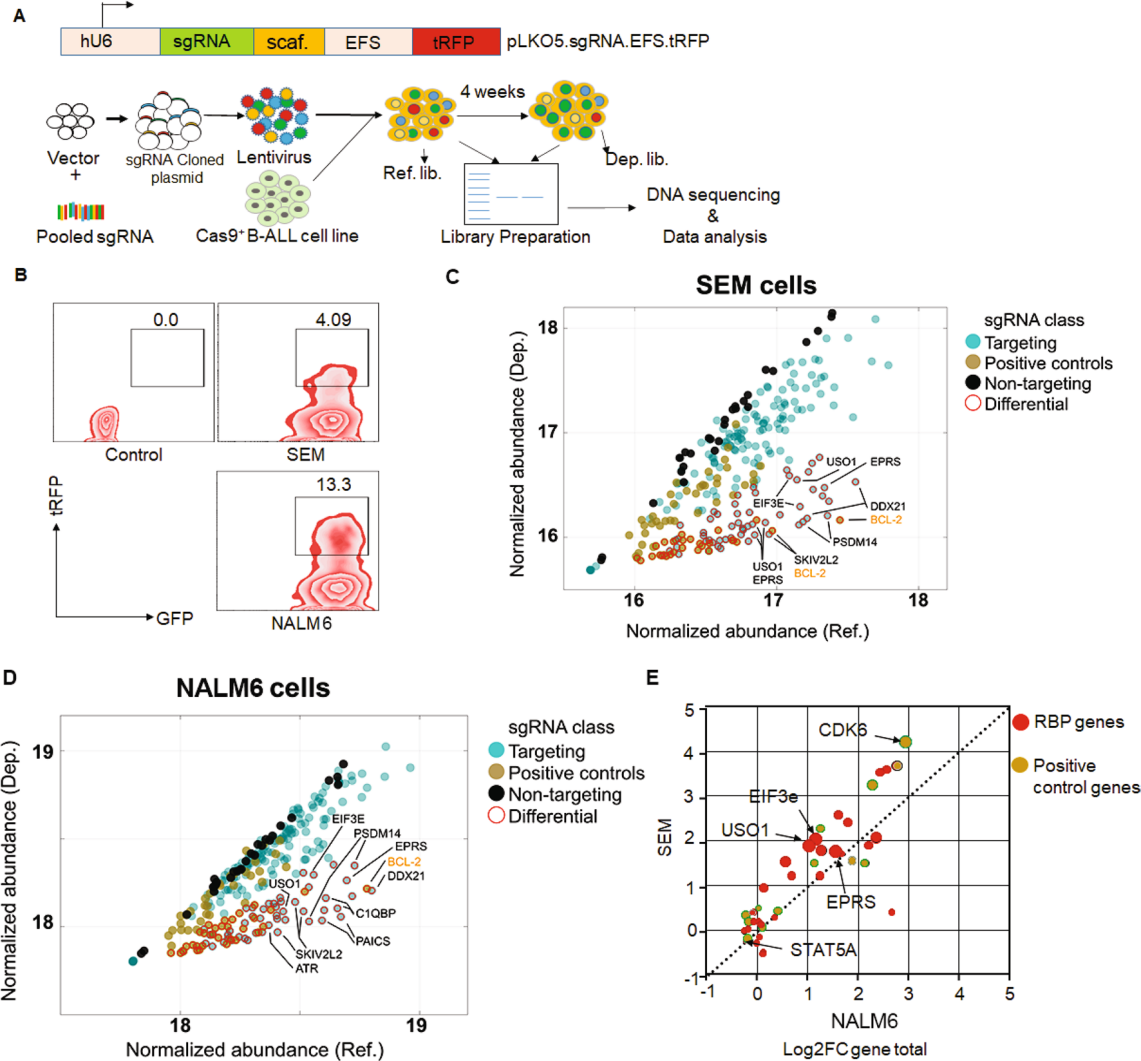
Sub-genomic CRISPR screen identifies functionally important genes in *MLL-AF4*-translocated B-ALL. (**A**) Schematic of sub-genomic CRISPR screen. (**B**) FACS contour plots reflecting sorting strategy based on high expression of Cas9 (GFP) and pooled guide RNA library (tRFP) in B-ALL cell lines. (**C**, **D**) Variance-stabilized normalized abundance for individual sgRNAs in Reference (Ref; x-axis) and Depletion (Dep; y-axis) libraries in SEM (C) and NALM6 (D) cell lines*.* Dots are colored by sgRNA class (dark yellow: positive controls; black: non-targeting negative controls; teal: targeting sgRNAs). Dots highlighted with a red border were classified as differentially abundant (Log2 fold change [Log2FC] > 1, Wald adjusted p value < 0.001). Genes with three or more differential sgRNAs are highlighted in the inset and colored by class. (**E**) Differential expression of sgRNAs aggregated by gene (Log_2_FC gene total), in NALM6 (x-axis) vs SEM (y-axis) cell lines. Dots in the upper left represent genes with a higher fold-change in SEM cells, while those on the lower right represent genes with a higher fold-change in NALM6 cells. Dots are colored by sgRNA class and sized according to the number of individual sgRNAs that showed significant differential representation in the Dep libraries.

Previously, we identified 36 putative RBPs that were significantly dysregulated in primary human *MLL*-rearranged B-ALL^15^. To directly query the functional relevance of RBP dysregulation in *MLL*-rearranged B-ALL, we performed a sub-genomic CRISPR screen (Fig. 1A). The library consisted of sgRNAs targeting 36 RBP genes, 12 “positive control” genes, representing known vulnerabilities in MLL-translocated leukemia^17^, and 28 non-targeting (NT) sgRNAs. The screen was designed using a two-vector lentiviral system. First, B-ALL cells with and without *MLL-AF4* translocation (SEM and NALM6, respectively) were stably transduced with a *Cas9-P2A-EGFP* transgene, followed by FACS sorting of the transduced cells based on GFP positivity. Next, Cas9-GFP^+^ SEM and NALM6 cells were transduced at a MOI of < 0.3 with the pooled sgRNA lentiviral library, comprised of 268 unique sgRNAs. Cells were subsequently FACS sorted 48 h following transduction for GFP and tRFP double positivity (Fig. 1B). 2 × 10^6^ cells were sorted, of which 10^6^ cells were used to isolate genomic DNA for the reference (REF) libraries and the remainder were cultured to maintain 3700 × coverage until collection of genomic DNA for the depletion (DEP) libraries following 28 days in culture. Experimental replicates of REF and DEP libraries showed a high degree of concordance in abundance of both individual sgRNAs and for total sgRNAs per gene (Supplementary Fig. 1D & 1E). In addition, the SEM and NALM6 REF libraries showed overall similar rates of individual gRNA incorporation, as measured by the abundance of each gRNA in each of the cell lines and biological replicates (Supplementary Fig. 1F). As expected, a majority of “positive control” genes, including *BCL2*, *COA5*, *CDK6,* and *MYC*, were significantly downregulated in the DEP libraries in both NALM6 and SEM cells. This is not surprising as many of these are known oncogenic genes, particularly in B-ALL. Non-targeting or “negative control” sgRNAs were consistently unchanged between the REF and DEP libraries (Fig. 1C, D). Comparing the results across cell lines, we found that sgRNAs targeting three genes, *USO1*, *EIF3E* and *EPRS* were significantly depleted in SEM cells (p < 0.001), when compared to NALM6 cells (Fig. 1E). Interestingly, sgRNA dropout in general was more readily observed in SEM cells than in NALM6 cells, potentially due to the fact that these genes were selected based on high expression in patient B-ALL samples with *MLL-AF4* translocation. Of these three genes, *USO1* had previously been detected in a genome-wide CRISPR screen as a vulnerability in MV-4–11 cells, which also harbor the *MLL-AF4* fusion gene^17^.

### USO1 is directly regulated by MLL-AF4

**Figure 2:**
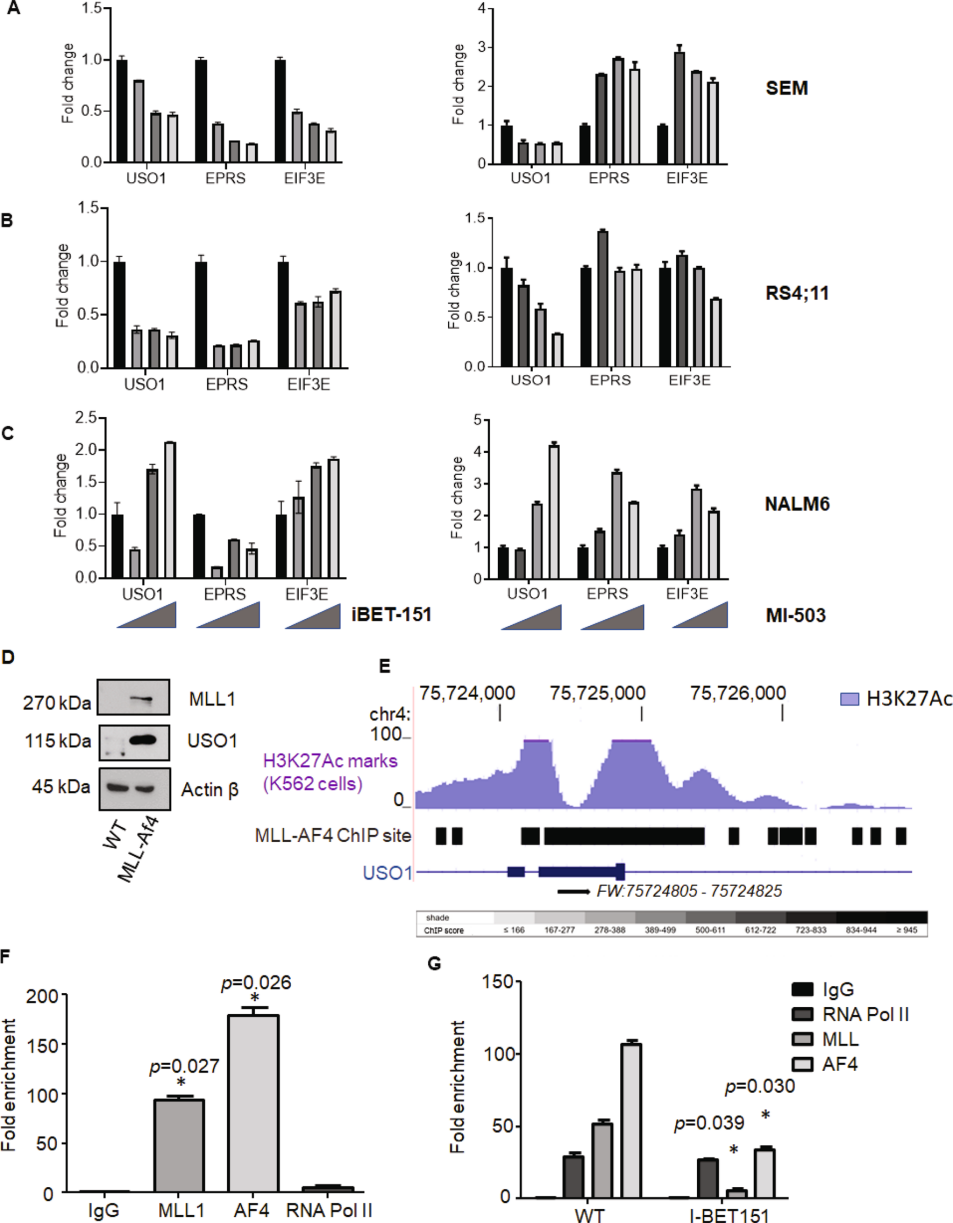
Dependence of RBP gene expression on MLL-AF4 translocation. (**A–C**) Effect of I-BET 151 and MI-503 treatment on mRNA expression levels of *USO1,EPRS* and *EIF3E*, measured by RT-qPCR, in SEM (**A**), RS4;11 (**B**), and NALM6 (**C**). The cells were treated with increasing concentrations of I-BET151 (DMSO only, 0.5, 1 and 2 μM) or with increasing concentrations of the menin inhibitor, MI-503 (DMSO only, 0.12, 0.25, and 0.5 μM). RT-qPCR was performed with an optimized set of primers, normalized to 18S, and then represented as fold-change from vehicle-treated control. **D**. Western blot analysis of murine bone marrow cells with and without transduction with MLL-Af4 (WT versus MLL-Af4), for MLL1 (top), USO1 (middle) and β-actin (lower). (**E**) UCSC genome browser shot of the *USO1* locus showing the *MLL-AF4* ChIP site(s), as identified from the ChIP-Seq data from Lin et al.^21^, in a gene expression regulatory region; *Courtesy: UCSC Genome Browser*. Shown are the H3K27Ac track in hematopoietic K562 cells (Blue), and MLL-AF4 binding sites represented as a grayscale score, with black indicating the highest score/highest number of reads from the dataset. (**F**) Chromatin immunoprecipitation with indicated antibodies (MLL1, AF4, and RNA Pol II), followed by qPCR (ChIP-qPCR) analysis for quantitation of bound *USO1* promoter/regulatory region to MLL1 and AF4 pulldown samples. Shown is the fold-enrichment for qPCR of the *USO1* regulatory site over background (t test; *P < 0.05) (**G**) SEM cells treated with 1 µM of I-BET151 for 48 h. and subjected to ChIP qPCR with MLL1 and AF4 antibodies as in (**F**) (t test; *P < 0.05).

*USO1*, *EPRS* and *EIF3E* expression was assessed in, SEM, RS4;11 (another *MLL-AF4* translocated cell line), and NALM6 (Supplementary Fig. 2A & 2B). To query their regulation by the MLL-AF4 fusion protein, we analyzed their expression following inhibition with I-BET151^18^, a BRD4 BET domain inhibitor, MI-503 menin-MLL1 inhibitor^19^ and EPZ5676, a DOT1L inhibitor^20^, all known to inhibit *MLL*-dependent gene expression regulation. With increasing doses of I-BET151, MI-503 and EPZ5676 there was a decrease in the *USO1* mRNA expression level in SEM (Fig. 2A), and RS4;11 cells (Fig. 2B), but not in NALM6 cells (Fig. 2C as well as Supplementary Fig. 2C, 2D & 2E), suggesting that *USO1* expression is *MLL-AF4* dependent. A consistent reduction in *EIF3E* and *EPRS* was not observed in the *MLL* translocated cell lines. We further queried the MLL dependence of *USO1* by over-expressing the MLL-Af4 transgene^21^ in murine bone marrow cells. Western Blot and RT-qPCR analysis showed upregulation of MLL1 and USO1 in MLL-Af4 transduced cells compared to control cells (Fig. 2D and Supplementary Fig. 2F). Using publicly available data^21^, we found that the *USO1* and *EIF3E* genes demonstrated multiple *MLL-AF4* binding sites within the 5’UTR, the first exon, and the first intron (Fig. 2E and Supplementary Fig. 2G). In contrast, the *EPRS* gene did not show strong *MLL-AF4* binding sites (Supplementary Fig. 2H). *EIF3e* and *EPRS* were previously reported to be “common essential genes”, per the depmap portal^22^, whereas *USO1* was not such a gene, suggesting its potential utility as a novel clinical target. To confirm our findings of USO1 dependence on *MLL-AF4*, we designed chromatin immunoprecipitation (ChIP) experiments, designing primers for the regulatory regions of *USO1* including the 5’ UTR within the first exon. We found significant enrichment of the *USO1* regulatory regions in both the MLL1 and AF4 pulldowns (Fig. 2F). Treatment of the cells with I-BET151 inhibited the association of both MLL1 and AF4 with the promoter region of *USO1* (Fig. 2G). Together, these findings indicate that *USO1* is a direct target of the *MLL-AF4* transcriptional program.

### *USO1* depletion alters B-ALL cell proliferation, survival and cell cycle

**Figure 3:**
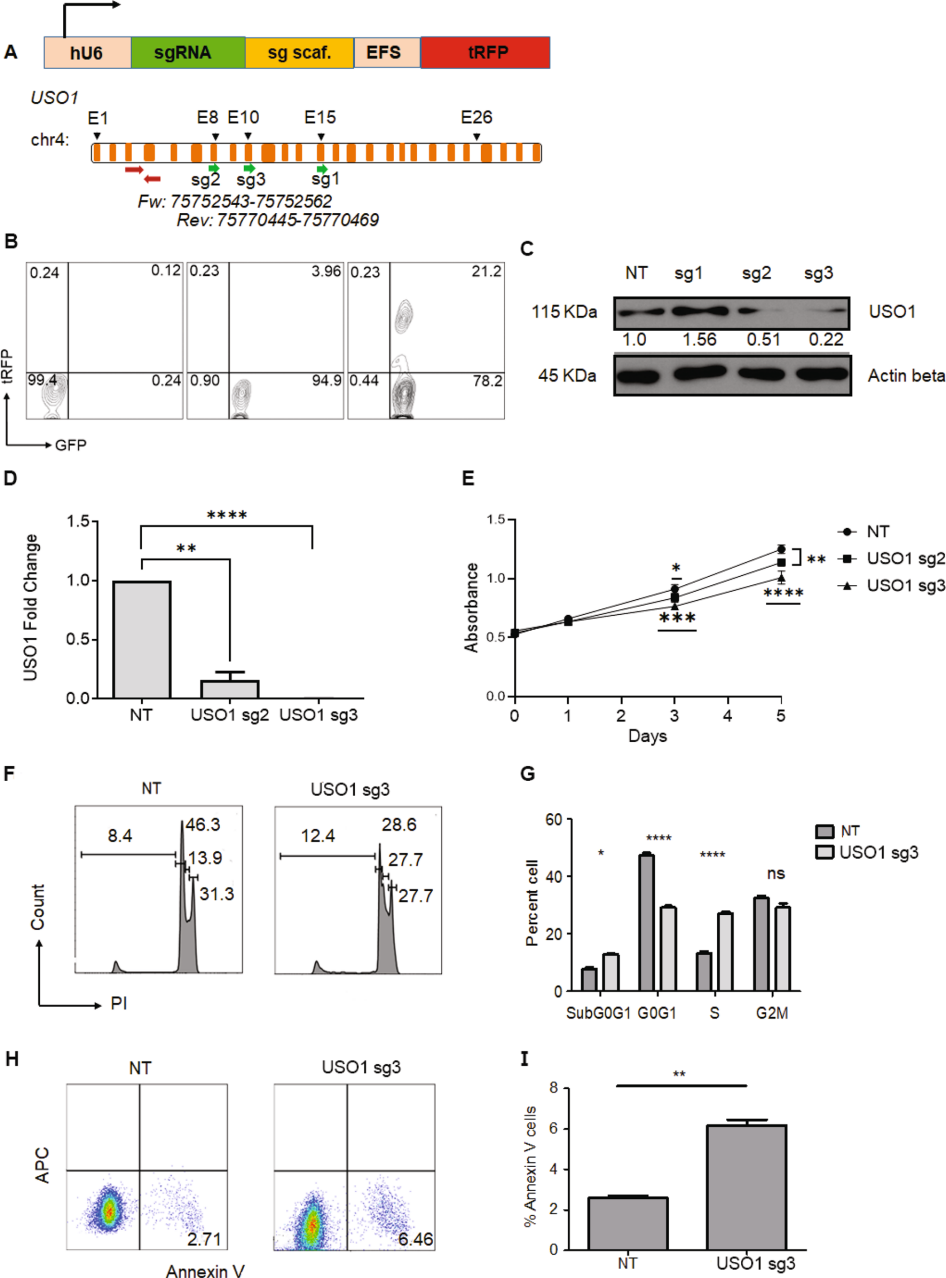
Depletion of USO1 leads to decreased cell growth, cell cycle arrest and increased apoptosis. (**A**) Schematic representation of the pLKO5.sgRNA.EFS.tRFP lentiviral vector. Abbreviations, hU6, human U6 promoter; sgRNA, short guide RNA; sg scaf, sgRNA scaffold; tRFP, turbo red fluorescent protein. (**B**) Sample FACS plots of SEM cells transduced sequentially with Cas9 vector and sgRNA containing vector. Left, non-transduced SEM cells; middle, transduced with pLentiCas9-GFP; right, cells transduced with both pLenti-Cas9-GFP and pLKO5 vector containing *USO1*-targeting sgRNA. (**C**) Western blot for USO1 in SEM cells following CRISPR/Cas9-mediated disruption of the *USO1* gene using three different sgRNAs (sg1-3) and NT, non-targeting sgRNAs. (**D**) RT-qPCR measurement of *USO1* in control (NT) and USO1 (sg2 & sg3) SEM cells (t test; **P < 0.01; ****P < 0.0001) (**E**) MTS assay to study the cell growth of USO1-depleted cells (sg2 & sg3), measured as Absorbance at 490 nM (t test; **P < 0.01; ***P < 0.001). (**F**, **G**) Cell cycle analysis using propidium iodide (PI) staining of control cells and USO1-depleted cells (**F**) and quantitation of cells from cell cycle analysis (Two-way Annova with Bonferroni correction; *P < 0.05; ****P < 0.0001). (**H**, **I**) FACS plots of Annexin V positivity in control versus USO1-depleted cells (**H**), Quantitation of cells with Annexin V positivity (t test; **P < 0.01) (**I**).

*USO1* expression is upregulated in several types of cancer including B-ALL with *MLL-AF4* translocations^23–25^. To characterize the functional role of *USO1* in B-ALL suggested by our CRISPR screen, we utilized the previously mentioned two-vector lentivirus system to transduce SEM cells with three different lentiviral constructs containing sgRNAs that target different regions of the *USO1* gene (Fig. 3A, B). We found that two sgRNAs (sg2 and sg3) caused a significant downregulation of *USO1* protein and mRNA expression by approximately 80% in SEM cells (Fig. 3C, D). Cas9-mediated frameshift mutation in *USO1* was confirmed using TIDE assay^26^ (Supplementary Fig. 3A). The lack of complete ablation was a reproducible finding in all cell lines we tested, as multiple single-cell cloning experiments failed to produce cells with complete knockout and multiple bulk cultures experiments showed retained partial expression of < 25%. Nonetheless, we observed that bulk *USO1*-depleted SEM cells from sg2 and sg3 showed reduced proliferation by MTS assay (Fig. 3E). Since sg3 showed near-total ablation of USO1 protein expression, we later used sg3 to target *USO1* in RS4;11 cells (Supplementary Fig. 3B & 3C), finding downregulation, but not complete depletion, of USO1 protein. Propidium Iodide (PI) based cell cycle analysis on *USO1*-depleted cells showed an increased percentage of cells in the G0/G1 stage, suggesting cell cycle arrest, and more cells in Sub-G0/G1, suggesting increased apoptosis (Fig. 3F, G). Increased cell death was also observed in the USO1-depleted cells by Annexin V staining (Fig. 3H, I). Interestingly, *USO1*-depleted cells treated with I-BET151 also showed increased cell cycle arrest and apoptosis (Supplementary Fig. 3D–3G), suggesting an additive effect with this inhibitor of BRD4. To confirm our findings in an orthogonal system, we introduced siRNAs targeting *USO1* using nucleofection. In these short-term assays, we found that there was partial reduction of *USO1* mRNA, as expected with siRNA-mediated knockdown (Supplementary Fig. 4A) and increased Annexin V staining (Supplementary Fig. 4B, C). There were also increased cells in Sub-G0/G1 and a modest reduction in cell proliferation as measured by the MTS assay (Supplementary Fig. 4E, F). Together, these observations confirm the importance of *USO1* in regulation of cell cycle and survival of B-ALL cells.

### *USO1* impacts pathways related to cellular proliferation and RNA homeostasis

**Figure 4:**
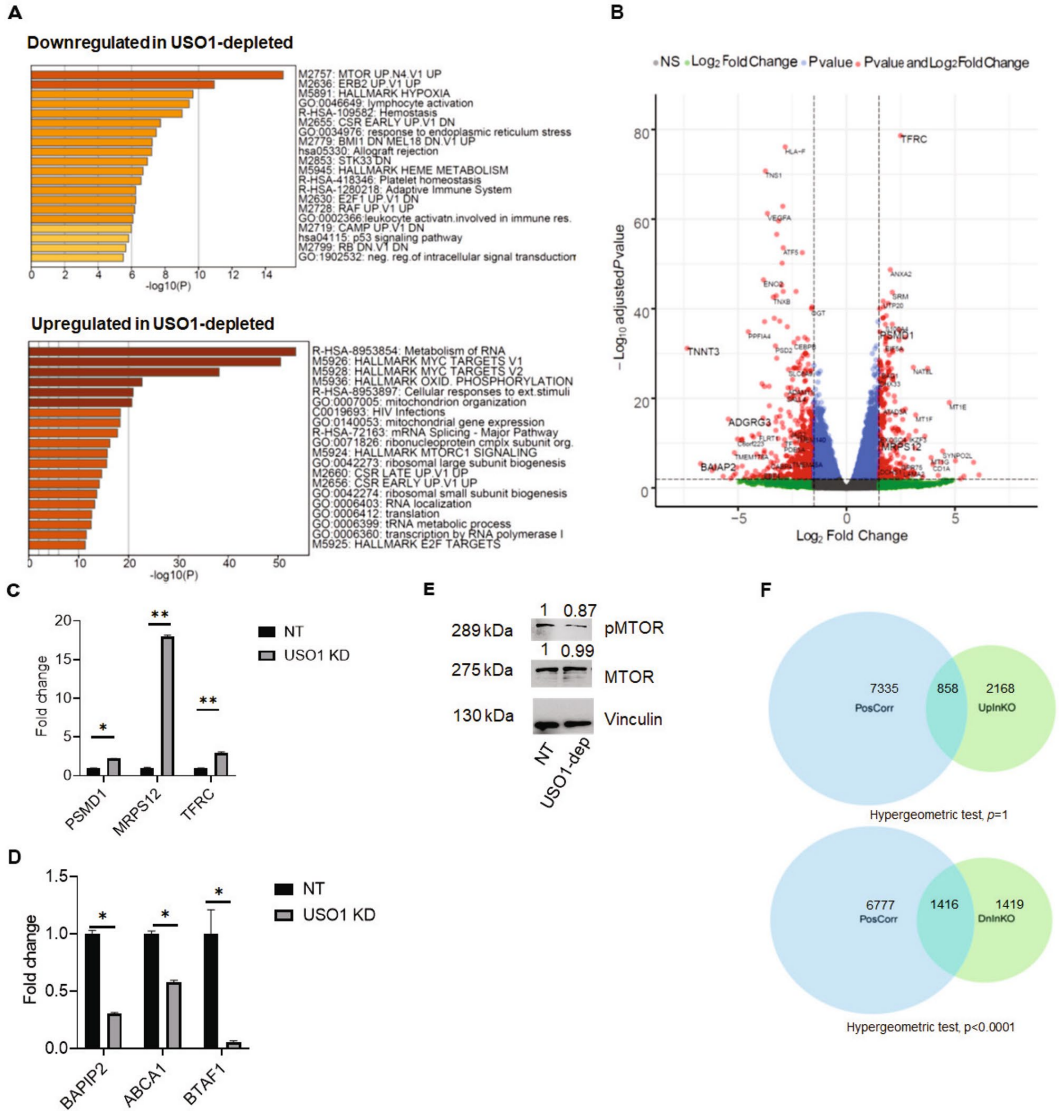
USO1 depletion significantly affects gene expression and pathways related to cell survival and proliferation. (**A**) Enrichment plots generated by the Metascape gene list enrichment analysis webtool on for the RNA-seq data from *USO1*-depleted versus NT control SEM cells. The top and bottom panels show pathways that are downregulated and upregulated in *USO1*-depleted SEM cells, respectively. (**B**) Volcano plot representing differentially expressed genes (*P*adj < 0.01 and Log2FC > 1.5) with several examples highlighted from the pathways enriched in (**A**). (**C**, **D**) RT-qPCR validation of differentially expressed genes identified in (**B**) (t test; *P < 0.05; **P < 0.01) (**E**) Western blot showing mildly reduced expression of p-MTOR (Ser2481) expression in USO1-depleted cells, compared to NT control, while MTOR remains unchanged. (**F**) Venn diagrams showing the number of shared genes between USO1-positively correlated genes in Target-Phase II ALL dataset with the genes that are significantly upregulated (top) or downregulated (bottom) in *USO1*-depleted SEM cells. A hypergeometric test was utilized to compare the overlaps between the datasets using a genome size of 24,278 genes. Total and shared number of genes are indicated.

To evaluate the effect of *USO1* depletion on the overall gene expression pattern in B-ALL cells, we performed RNA-Seq on SEM cells that were depleted of USO1 by CRISPR/Cas9 (three biological replicates per condition). Following differential expression analysis of USO1-depleted and control cells, we utilized the Metascape algorithm and Gene set enrichment analysis (GSEA) to assess pathway enrichment^27,28^. We found that significantly downregulated pathways included MTOR, ERB2, and Hallmark Hypoxia, while upregulated terms included Metabolism of RNA and Hallmark MYC targets (Fig. 4A and Supplementary Fig. 5A). Analysis of Gene Ontology -Molecular Function gene sets revealed positive enrichment of several gene sets related to RNA homeostasis in the USO1-depleted cells (Supplementary Fig. 5B). Selected examples of up- and down regulated genes (log2FC > 1.5 and *P*adj. value < 0.01), from the enriched pathway identified by GSEA are highlighted in the Volcano plot for RNA-seq data (Fig. 4B). RT-qPCR was used to confirm these same significantly upregulated (*TFRC*, *MRPS12,* and *PSMD1)* (Fig. 4C) and downregulated genes (*BAPIP2, ABCA1* and *BTAF1*) (Fig. 4D) in USO1-depleted SEM cells. This led us to hypothesize that there were alterations in expression and activation of MTOR in USO1-depleted cells using western blotting. We observed there was a mild downregulation of p-MTOR (Ser2481) in the USO1-depleted cells (Fig. 4E). Hence, it appears that *USO1* regulates several pathways that are known to play a role in cell survival and cell death^29^.

To assess whether the gene expression changes caused by *USO1*-depletion had any clinical relevance, we turned to the Target Phase II Acute lymphoblastic leukemia dataset, accessed via the cBioPortal interface^30,31^. We calculated correlation coefficients between *USO1* and 24,278 genes detected by RNA-seq in 203 samples from 154 patients. Using a cut-off q value of 0.001, we then overlapped genes up- or down-regulated in USO1-depleted with genes that had either a positive or negative correlation co-efficient. We found a highly significant overlap between genes that were positively correlated with *USO1* across the B-ALL samples and downregulated in USO1-depleted cells (Hypergeometric test; p < 0.0001), but not in genes that were upregulated in USO1-depleted cells (non-significant p value) (Fig. 4F, Supplementary 5C & 5D). This suggests that USO1 regulates gene expression in patient samples of B-ALL. As concrete examples, differentially expressed genes confirmed by RT-qPCR (Fig. 4C, D) showed a direct correlation with USO1 expression in the ALL dataset from cBioPortal (Supplementary Fig. 6A–6D). These findings strongly suggest that *USO1* plays a role in gene regulation in human B-ALL.

### USO1 inhibits the MLL-AF4-driven leukemogenesis in primary murine bone marrow cells

**Figure 5:**
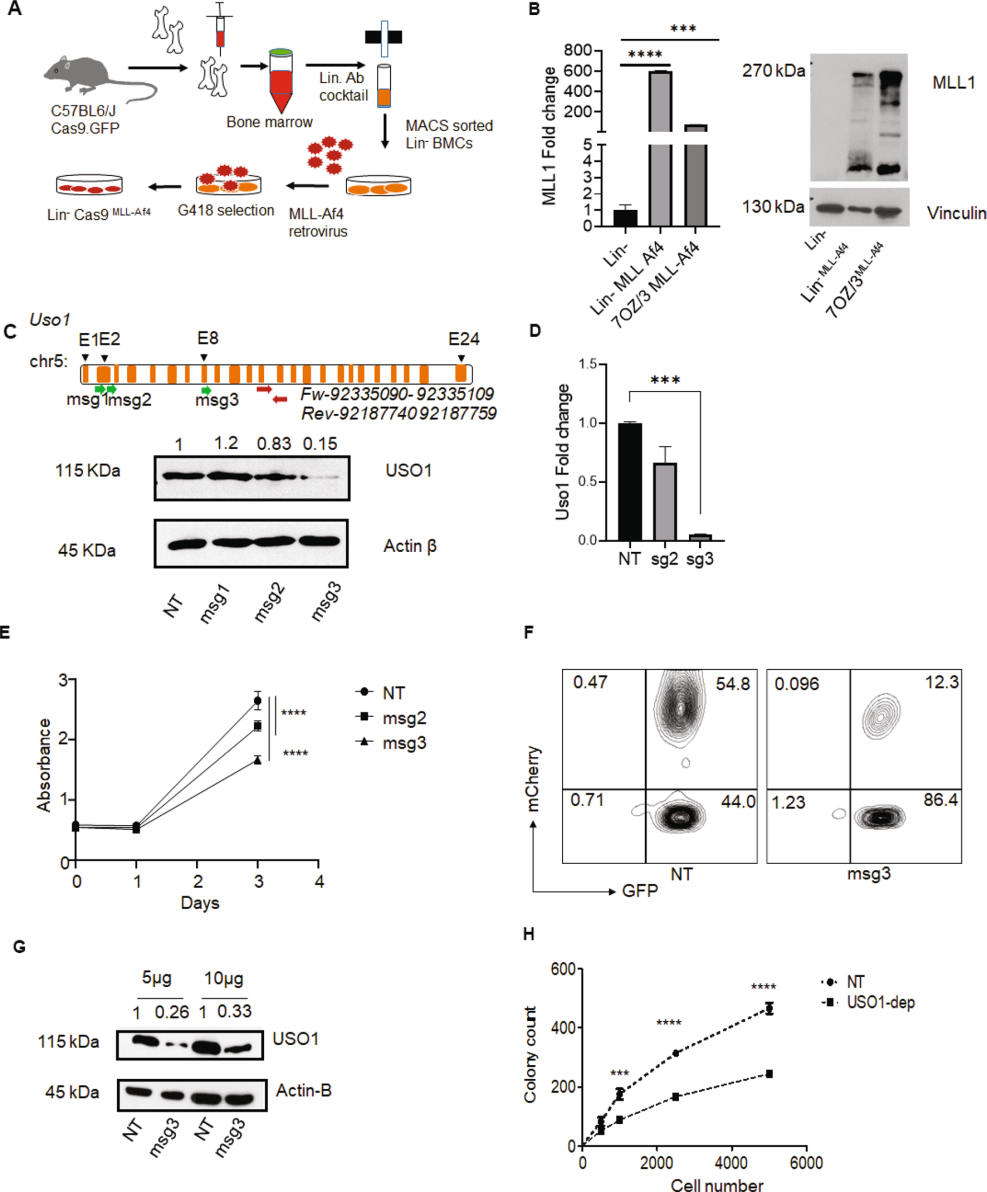
USO1 depletion in transformed bone marrow cells shows reduced proliferation and colony forming potential. (**A**) Schematic of an in vitro model system to transform Lin^-^ bone marrow cells from *Cas9-egfp* mice using overexpression of *MLL-Af4* transgene in. (**B**) Analysis of overexpression of MLL-Af4 by RT-qPCR and western blot in retrovirally transduced Lin^-^Cas9^MLL-Af4^ cells, Lin^-^Cas9 cells were used as negative control and 70Z/3 cells transduced with *MLL-Af4* were used as positive control. RT qPCR was performed with an optimized set of primers, normalized to L32, and represented as fold-change from an internal control for each experiment. Western Blotting was performed with an antibody to MLL1. Vinculin was used as a high molecular weight loading control. (**C**) Upper panel, schematic of murine *Uso1* depletion experiments, showing location of sgRNAs relative to the gene, and RT-qPCR primer location. Bottom panel, western blot analysis of 70Z/3 cells transduced with three different sgRNAs targeting *Uso1* (msg1-3) cloned in the MSCV.sgRNA.mCherry.v1 vector. (**D**) RT-qPCR analysis of *Uso1* depletion in 70Z/3 cells. (**E**) MTS assay (Absorbance at 490 nm) to measure proliferation in *Uso1*-depleted 70Z/3 cells compared to NT control cells. (**F**) FACS plot showing gating schema to sort the Lin^-^Cas9^MLL-Af4^ GFP^+^ mCherry^+^ population following transduction with the *Uso1* msg3 vector. (**G**) Western blot was used to confirm the reduction in USO1 expression in sorted Lin^-^Cas9^MLL-Af4^ cells. (**H**). Colony formation assay using Lin^-^Cas9^MLL-Af4^ cells in methylcellulose assay as described in methods with titration of input cell number (t test, ***P < 0.001; ****P < 0.0001).

Having demonstrated that *USO1* is required for the survival and growth of *MLL-AF4*^+^ cells in culture, we wanted to determine if *USO1* was required in an experimentally induced primary cell model of *MLL-AF4*-driven leukemia. Briefly, Lin^-^ cells from the bone marrow of *Cas9-EGFP* mice^32^ were transduced with MLL-Af4 retrovirus^33^ (Fig. 5A) and selected with G418. We confirmed expression of the MLL-Af4 transgene, finding overexpression by RT-qPCR and western blot analysis (Fig. 5B). As expected, we observed rapid proliferation and expansion of the Lin^-^ *Cas9*^*MLL-Af4*^ cells. In order to deplete *Uso1* from these cells, we designed and cloned three *Uso1* murine specific sgRNAs (msg2 and msg3) into our internally designed MSCV.EFs.mCherry retroviral vector. To determine their effectiveness, we sorted GFP^+^ mCherry^+^ 70Z/3 cells transduced with the MSCV retroviruses, briefly expanded them in culture, and then queried USO1 expression. Western blot and RT-qPCR demonstrated that msg2 and msg3 both resulted in significant reduction of USO1 protein and mRNA expression (Fig. 5C, D). *Uso1*-depleted 7OZ/3 cells also had reduced cell growth by MTS assay which correlated with the different levels of USO1 depletion for msg2 and msg3 (Fig. 5E). From these data, the msg3 retrovirus was selected for transduction of Lin^-^ *Cas9*^MLL-Af4^ cells. After sorting GFP^+^ mCherry^+^ transduced Lin^-^ *Cas9*^MLL-Af4^ cells (Fig. 5F), USO1-depletion was confirmed by western blot, in which protein expression was reduced to 26% (Fig. 5G), and the cells were subsequently used in a colony formation assay. Functionally, USO1-depletion resulted in significantly fewer colonies in Lin^-^*Cas9*^MLL-Af4^ cells compared to NT control cells at 12 days. This change was maintained at several different starting numbers of Lin^-^*Cas9*^MLL-Af4^ cells (Fig. 5H). Hence, the function of *USO1* is preserved in not only in human B-ALL cell lines but also in primary murine MLL-Af4 transformed cells.

## Discussion

The molecular mechanism of *MLL-AF4* driven leukemogenesis remains incompletely understood, and this subtype of B-ALL is highly aggressive^5,7^. Although there are several fusion partners for *MLL* in acute leukemia, downstream transcriptional dysregulation is a common feature^15,34,35^. In this study, we sought to understand whether overexpression of putative RBPs, which we identified previously, contributes to the pathogenesis of *MLL-AF4*^+^ B-ALL^15^. We performed a focused sub-genomic CRISPR/Cas9 dropout screen to specifically address whether these putative RBPs had a functional role in leukemia cell growth. Indeed, we identified three genes (*EIF3E, EPRS,* and *USO1* ) that appeared to be required in the *MLL-AF4*^+^ cell line, SEM. These genes showed slightly higher rates of dropout in SEM cells than in NALM6 cells. Of these, *USO1* expression showed a dependence on *MLL-AF4*, whereas *EIF3E* and *EPRS* did not show the same dependence and were previously reported to be “common essential genes”, per the depmap portal^22^. Follow-up studies confirmed a role for USO1, both in cell lines and in a model of *MLL-AF4* driven leukemia in primary murine bone marrow cells. RNA sequencing revealed that USO1 regulates numerous pathways, including mTOR, MYC targets, as well as elements of RNA homeostasis.

One of the challenges of genomic-scale CRISPR screens is that genes with a small average effect size on the phenotype of interest can be quite difficult to identify and can frequently be “drowned out” by genes with a larger effect size^36^. As we were particularly interested in the role that RBPs play in B-ALL, and their effect size in cell lines is unknown, we chose to perform a sub-genomic essentiality screen targeting a pre-defined set of RBPs known to be highly expressed in B-ALL. Therefore, under the hypothesis that a significant proportion of the sgRNAs would have a negative effect on cell viability, our design included a substantial number of both positive and negative control sgRNAs in order to properly model the null effect distribution and compare the effect size of functional RBPs to that of known essential genes. Secondly, the use of a non-*MLL-AF4* control cell line allowed us to identify those proteins whose expression might be most important in *MLL-AF4* leukemia. Supporting the idea that such specific effects may not be seen in genome scale studies, *USO1* dropout was detected in one prior genome-scale analysis, whereas it was not observed in two other genome scale studies that queried vulnerabilities in *MLL*-translocated leukemia^17,37,38^. Thus, our approach to perform this type of CRISPR/Cas9 screen may help inform the design of future forward genetic screens.

Our group is interested in understanding RNA binding proteins in B-ALL, and we recently described the functional role of *IGF2BP3* in pathologic expansions of cells within the hematopoietic system and its requirement for survival and growth in B-ALL cell lines^15^. Here, we focused on *USO1*, based on its identification in this screen as well as a prior study in MV-4–11 AML cells that had identified *USO1* as a factor required for AML survival^17^. A recent study reported a *KMT2A(MLL)-USO1* fusion gene in a secondary AML, hinting at a further connection between *MLL*-driven leukemia and *USO1*^39^. More generally, USO1 has been shown to be of functional importance in cancer^23–25^. USO1 was recently reported to have RNA-binding function in studies utilizing high-throughput biochemical techniques^16,40,41^, despite a canonical role in the regulation of vesicular transport^42,43^. Adding to these prior descriptions of USO1 function, we validated *USO1* as a MLL-AF4-induced gene, and found that it was functionally required in cell lines and in primary bone marrow for MLL-AF4 dependent phenotypes. This work firmly establishes the significance of this protein in acute leukemia, which was not previously appreciated.

Recent studies in multiple myeloma have shown that USO1-deficient cells showed have reduced cell proliferation and increased apoptosis via regulation of Erk pathway activity^24^. Transcriptome analysis of *USO1*-depleted SEM cells in our study demonstrated a mixed picture, with the downregulation of certain cancer and cell growth-related pathways, including *mTOR* and *ERB2*, but concurrent downregulation of RNA metabolism and MYC targets. Curiously, there was also upregulation of the mTORC1 hallmark pathway in USO1-depleted cells (as opposed to downregulation of mTOR generally), perhaps indicating a specific effect on mTORC2. Nonetheless, we observed decreased phospho-mTOR, which is consistent with the effect seen on cell growth and cell cycle. Additionally, our data suggests that USO1 is associated with other molecular functions of gene regulation, such as RNA homeostasis. Interestingly, the pathways noted to be deregulated show similarity to those deregulated upon inhibition of the RNA demethylase FTO^44^. It is important to note, however, that we have not characterized its function as a RBP. It is tempting to speculate that USO1 is a bifunctional protein with roles in vesicular transport and RNA binding, perhaps in a manner similar to *YBX1*^45^. YBX1 appears to bind to and sort microRNAs, specifically, miR-223, into exosomes. By regulating this process, YBX1 can impact cellular homeostasis. Hence, further work to assess the molecular role of USO1 as a putative RBP in *MLL-AF4* translocated leukemia is warranted.

Overall, our study successfully queried the functional relevance of a set of genes identified from primary patient samples using expression profiling. Here, we provide a rubric for how to functionally analyze a prioritized list of genes in leukemogenesis, or in other pathogenetic processes. In addition, we establish a role for the putative RBP, USO1, in leukemogenesis. Given the broader range of cancer types that show *USO1* dysregulation, our work may have implications beyond those in B-ALL. Furthermore, understanding how non-canonical RBP may participate in leukemogenesis may open up new avenues in developing novel strategies for the diagnosis, prognosis, and treatment of B-ALL.

## Methods

### Cell lines and cell culture

All the cell lines involved in the study were maintained at 37 °C in a humidified incubator at 5% CO_2._ RS4;11 (ATCC CRL-1873), NALM6 (ATCC CRL-3273) were cultured in RPMI 1640 supplemented with 10% FBS. 70Z/3 (ATCC TIB 158) cells were cultured in RPMI 1640 supplemented with 10% FBS and 0.05 mM 2-mercaptoethanol. SEM cells (DMZ-ACC 546), MV-4–11 (ATCC CRL-9591) were cultured in Iscove’s Modified Dulbecco’s Medium (IMDM) supplemented with 10% FBS. Mouse bone marrow derived Lineage (Lin^-^) cells were cultured in IMDM media supplemented with 15% FBS, 20 ng/mL mTPO, 20 ng/FLT3 ligand and 50 ng/mL mSCF.

### Sub-genomic CRISPR screen

A sub-genomic CRISPR/Cas9 screen was designed to target 36 RBP genes and 12 “positive control genes”, and included 28 negative control (or non-targeting) single guide RNAs (sgRNAs). The positive control genes, representing known vulnerabilities in MLL-translocated acute leukemia, were selected from top 100 genes dysregulated in Genome wide CRISPR screen in MV-4–11 cells^17^. These genes are expected to “drop out” in a CRISPR screen of MLL-translocated leukemia, but it is not known whether they are specific for MLL-translocated leukemia. This design was adapted to provide enough non-targeting controls in the context of a sub-genomic screen, where a significant proportion of targeting sgRNAs may be expected to change. Five sgRNAs were designed for each RBP or positive control genes, using sgRNA design tools from Broad Institute^46^. pLKO5.sgRNA.EFS.tRFP is a lentiviral vector, which contains EF-1 alpha binding sequence (EFS) upstream of tRFP, was obtained from Addgene (#57,823)^47^. The 268 pooled sgRNA were then cloned into pLKO5.sgRNA.EFS.tRFP lentiviral vector using standard protocols^17^. Prior to CRISPR/Cas9 screening, B-ALL cell lines with *MLL-AF4* translocation (SEM)^33^ and without *MLL-AF4* translocation (NALM6)^48^ were stably transduced with pLentiCas9-GFP^49^ lentivirus and sorted on GFP positivity, with subsequent confirmation of Cas9 expression (Supplementary Fig. 1A). pLKO5.sgRNA.EFS.tRFP lentiviral pool titers were calculated from SEM and NALM6 cell transduction. For experiments, bulk GFP^+^ SEM and NALM6 cells were infected at < 0.3 MOI and 2 × 10^6^ cells GFP^+^ tRFP^+^ were sorted by FACS after 48 h of infection (Supplementary Fig. 1B). Genomic DNA (gDNA) isolated from 10^6^ cells was used for construction of the Reference (REF) library sample, and the other 10^6^ cells were cultured and expanded. Cells were split every five days and 10^6^ cells were reseeded for culture^50^ to maintain a sgRNA representation of 3700X. Following 28 days of culture, cells were harvested and gDNA was isolated for the Depletion (DEP) library sample preparation.

### Library preparation, DNA sequencing, and analysis

Sequencing libraries were prepared from both the Reference (REF) and Depleted (DEP) genomic DNA (gDNA) samples obtained at days 0 and 28 of CRISPR screen experiment, respectively^17,50^. Libraries were prepared from 200 ng of input DNA, by using Q5 high-fidelity DNA polymerase (#M0492S, NEB) and Illumina adapted primers to amplify the sgRNA target region from the gDNA, as previously described^17^ (Supplementary Fig. 1C). The purified PCR product was quantified using Qubit and quality control was done using Bioanalyzer and sequenced on HiSeq 3000 at the Technology Center for Genomic and Bioinformatics at UCLA. Adapter sequences were removed using in-house scripts. Candidate reads (those containing a valid primer sequence and with a minimum length of 20 bp after trimming) were aligned to the sgRNA library using bowtie v0.12.8^51^ with a maximum tolerance of one mismatch. Counts tables for both individual sgRNAs and gene-level summaries were compiled from non-ambiguous hits for both the Reference and Depletion libraries in each experiment and for each cell line. Count tables were processed with DESeq2^48^ to obtain variance-stabilized normalized abundance and rank sgRNAs and genes based on differential abundance (moderated fold change and adjusted Wald test p value).

### Cell line treatment with transcription inhibitors

B-ALL cell lines were plated at 0.5 × 10^6^ cells/mL density 24 h before treatment, and harvested 48 h after initiating treatment with the chemical inhibitor. I-BET151, a BRD4 inhibitor was reconstituted in DMSO (10 mM), was diluted in complete media and added to the cells at a concentration of 0.5 µM, 1.0 µM and 2.0 µM. MI-503, a menin-MLL inhibitor, was used to treat the cells at 0.12 µM, 0.25 µM and 0.5 µM. EPZ5676, a DOT1L inhibitor, was used to treat cells at a concentration of 0.5 µM of EPZ5676.

### Cell proliferation, cell cycle and apoptosis assays

Cell proliferation assay was performed using standard MTS assay protocol. 5000 cells were plated in 100 µL volume of media in a single well of 96-well tissue culture plates. Cells were harvested at different time points of day 0, day1, day 3 and day 5. MTS reagent mix was prepared by adding 100 µL of PMS solution (0.21 mg/mL) to 1 ml of MTS reagent (0.33 mg/mL) and 20 µL reagent mix was added to each well. The plate was incubated at 37 °C for 2 h and absorbance was taken at 490 nm in a microplate reader.

Cell cycle analysis was performed using propidium iodide (PI). Cells were harvested and washed in PBS and fixed in 70% ethanol overnight at − 20 °C. Fixed cells were washed with PBS and centrifuged at 2500 rpm. PI solution (2 mg/10 mL) was diluted in PBS and added with 0.2 mg/mL of DNase free RNase A. Nearly, 300 µL of the PI solution was added to each tube and incubated at RT for 2 h. The stained samples were analyzed by flow cytometer.

Annexin V staining was performed to study the apoptosis in the cells using standard protocol. Briefly, cells treated with inhibitors/siRNAs were harvested and washed in PBS before resuspending in binding buffer (10^6^/mL). 100 µL of cell suspension was stained with 0.5 µL of Annexin V antibody conjugated to Pacific blue and incubated at RT for 30 min. After incubation, 300 µL of binding buffer was added to the sample and analyzed by flow cytometer.

### siRNA knockdown of cell lines

siRNA transfection was performed using standard Nucleofection program provided by the manufacturer. SEM cells were Nucleofected using the 4D Nucleofector System (Lonza, Cologne, Germany). Cells were washed with phosphate-buffered saline and then resuspended in nucleofection solution (SF Cell Line 4D-Nucleofector X Kit, Lonza, Cologne, Germany), at a final concentration of 2 × 10^6^ cells/100 µL reaction. Cells were nucleofected with 30 pmol of control siRNA, USO1 siRNA1, or USO1 siRNA 4, in 100 µL cuvettes using program CV-104. Immediately after nucleofection, 500 µL of pre-warmed, antibiotic-free media was added to the cuvette and incubated for 10 min at RT. After incubation cells were transferred to a 12 well plate containing 1.5 mL of media. Nucleofected cells were maintained at 37 °C and 5% CO_2_ prior to harvesting for analysis.

### RT-qPCR assays

Previous protocols were adapted for RT-qPCR, based on our prior work^15^. A full list of RT-qPCR primers is presented in Table S2. For normalization, we utilized RT-qPCR primers for 18S (human) and L32 (mouse).

### Western Blotting

Western Blotting was performed as previously described^15^. The blots were developed and imaged on ECL film or on a Bio-Rad Chemidoc digital imager using Super signal West Pico PLUS chemiluminescent reagent. EPRS (#A303-957A), EIF3E (#A302-984A), USO1 (#A304-513A) antibodies were purchased from Bethyl laboratories. USO1 (13,509–1-AP) antibody to detect mouse USO1 was purchased from Proteintech. MTOR (#2972 s) and Phospho-MTOR (Ser2481) (#2971) antibodies were procured from Cell Signaling Technology. Vinculin (Santa Cruz Biotechnology, # sc-73614) and Anti-β-Actin (Sigma Aldrich, #A1978) were used for loading controls.

### Chromatin immunoprecipitation (ChIP)

SEM cells were cultured with and without I-BET151 (Sigma Aldrich,# SML0666) and DMSO for 48 h at 37 °C^52^. ChIP was performed using EZ-Magna ChIP kit (Millipore, # 17–408) with MLL1 antibody (Bethyl laboratories, #A300-374A) and AF4 antibody (Abcam, #ab31812). The purified DNA was used as input for qPCR and binding was quantitated as previously described^52^.

### Immortalization of Lin^-^ bone marrow cells

All mice used in this study were obtained from Jackson Labs and were genotyped according to JAX protocols and maintained in the UCLA Division of Laboratory Animal Medicine. Bone marrow cells from C57BL6/J *Cas9-EGFP* mice^53^ (Jackson Laboratories, # 026,179) were isolated by flushing the bones from the mice and creating a single cell suspension. Cells were incubated with a lineage antibody cocktail and depleted for Lineage^+^ cells using MACS technology (Miltenyi Biotech). Lin^-^ cells were spin-infected and transduced with *MLL-Af4* retroviral preparation^33^. The MSCV-MLL-flag-Af4 plasmid was the kind gift via MTA by Dr. Michael Thirman (University of Chicago, Department of Medicine). After four rounds of transduction, cells were selected in 400 µg/mL G418 supplemented media for 7 days.

### MSCV sgRNA vectors

We generated a novel MSCV vector that can overexpress an individual sgRNA in addition to an mCherry reporter. In brief, the MSCV.mU6.sgRNA-EFs.mCherry.v1 retroviral vector was constructed by replacing a 2.1 kb EGFP-PGK.Puro fragment from the pMGP vector with a 2.7 kb sequence containing mU6.BbsI-stuffer-BbsI-scaffold-spacer-EFs.mCherry via BglII/ClaI digest. The sgRNA scaffold and EF-1α short (EFs) promoter elements were derived from the pLentiCRISPRv2 vector. The mU6 promoter was designed from the GenBank sequence NC_000076.6 (nt 79,908,880–79,909,195). A silent mutation was incorporated into the mCherry reporter element to remove an internal BbsI restriction site. The 1.2 kb stuffer sequence was derived from portions of the 1.8 kb firefly luciferase gene. The sgRNA sequences targeting mouse were designed as above and directionally cloned between the mU6 promoter and sgRNA scaffold sequence via BbsI. Detailed methods and vector maps are available upon request.

### Colony forming unit assay

A colony forming unit assay was performed to study the effect of USO1-depletion on the colony forming potential of Lin^-^Cas9^MLL-Af4^ cells. The assay was performed using the Methocult colony forming media (STEMCELL Technologies, #M3434)^54^. Briefly, approximately 5,000 Lin^-^Cas9^MLL-Af4^ USO1 depleted cells were mixed in 3.2 ml of overnight thawed Methocult media and plated in two 35 mm dishes along with the NT controls and cultured for 12 days. After 12 days of culture, individual 35 mm dishes were counted for both total number and morphologic subtypes of colonies formed by USO1 depletion and NT control cells.

### RNA-Seq library preparation and analysis

Libraries for RNA-Seq were prepared with Nugen Universal plus mRNA-Seq Kit to generate strand-specific RNA-seq libraries. Sequencing was performed on Illumina HiSeq 3000 SR 1 × 50 bp run. Data quality check was done on Illumina SAV. Demultiplexing was performed with Illumina Bcl2fastq2 v 2.19.1.403 program. The STAR ultrafast universal RNA-seq aligner v2.7.0d^55^ was used to align the reads to a genome index that included both the genome sequence (GRC38 human primary assembly) and the exon/intron structure of known human gene models (Gencode v29 genome annotation). Alignment files were used to generate strand-specific, gene-level count summaries with STAR's built-in gene counter. Independent filtering was applied as before^56,57^: genes with less than 6 total counts across all samples, count outliers, or low mappability (< 50 bp) were filtered out for downstream analyses. Expression estimates were computed in units of fragments per kilobase of mappable length and million counts (FPKMs). Differential expression analyses between *USO1* depletion and non-targeted controls was performed with DESeq2^27^ and genes were ranked based on moderated fold change and adjusted Wald test p value. Functional enrichment for selected genes was performed with Metascape^36^.

### Approvals and Compliance

This study was carried out in compliance with the ARRIVE guidelines. All animal experiments were carried out in accordance with relevant guidelines governing the use of animals in research. In addition, all experiments involving animals were approved by the University of California, Los Angeles, Chancellor’s Animal Research Committee (ARC), which was established for compliance with Public Health Service (PHS) guidelines on animal research.

## Supplementary information

Supplementary Information.

## Data Availability

All sequencing data have been deposited in the Sequence Read Archive (PRJNA658354). All research materials will be made available in accordance with UCLA policy.
