# Supplementary material for: Focused CRISPR-Cas9 genetic screening reveals USO1 as a vulnerability in B-cell acute lymphoblastic leukemia
Source: Sci Rep. 2021 Jun 23;11:13158. doi: 10.1038/s41598-021-92448-w (PMC8222245; doi:10.1038/s41598-021-92448-w)
Supplement: Supplementary file 1 — Supplementary Information. [file 41598_2021_92448_MOESM1_ESM.pdf]

SUPPLEMENTARY MATERIAL

Focused CRISPR-Cas9 genetic screening reveals USO1 as a vulnerability in B-cell acute lymphoblastic leukemia

Amit Kumar Jaiswal[1], Hellen Truong[1], Tiffany M Tran[1,2], Tasha L Lin[3], David Casero[4], Michael O. Alberti[5], and Dinesh S. Rao, M.D., Ph.D.[1,6,7]

[1] Department of Pathology and Laboratory Medicine, UCLA

[2]Molecular, Cellular and Integrative Physiology Graduate Program, UCLA

[3]Department of Internal Medicine, Division of Hematology/Oncology, UCLA

[4] Inflammatory Bowel and Immunobiology Institute, Cedars Sinai Medical Center, Los Angeles, CA 90048, USA

[5]Department of Pathology, Washington University, St. Louis

[6] Jonsson Comprehensive Cancer Center, UCLA

[7] Broad Stem Cell Research Center, UCLA

Corresponding Author:

Dinesh S. Rao, M.D., Ph.D.,
Associate Professor
Department of Pathology and Laboratory Medicine
David Geffen School of Medicine at UCLA
650 Charles E Young Drive, 12-272 Factor
Los Angeles, CA 90095
Tel. 310-825-1675
Fax. 310-825-0814
Email drao@mednet.ucla.edu

Figure S1

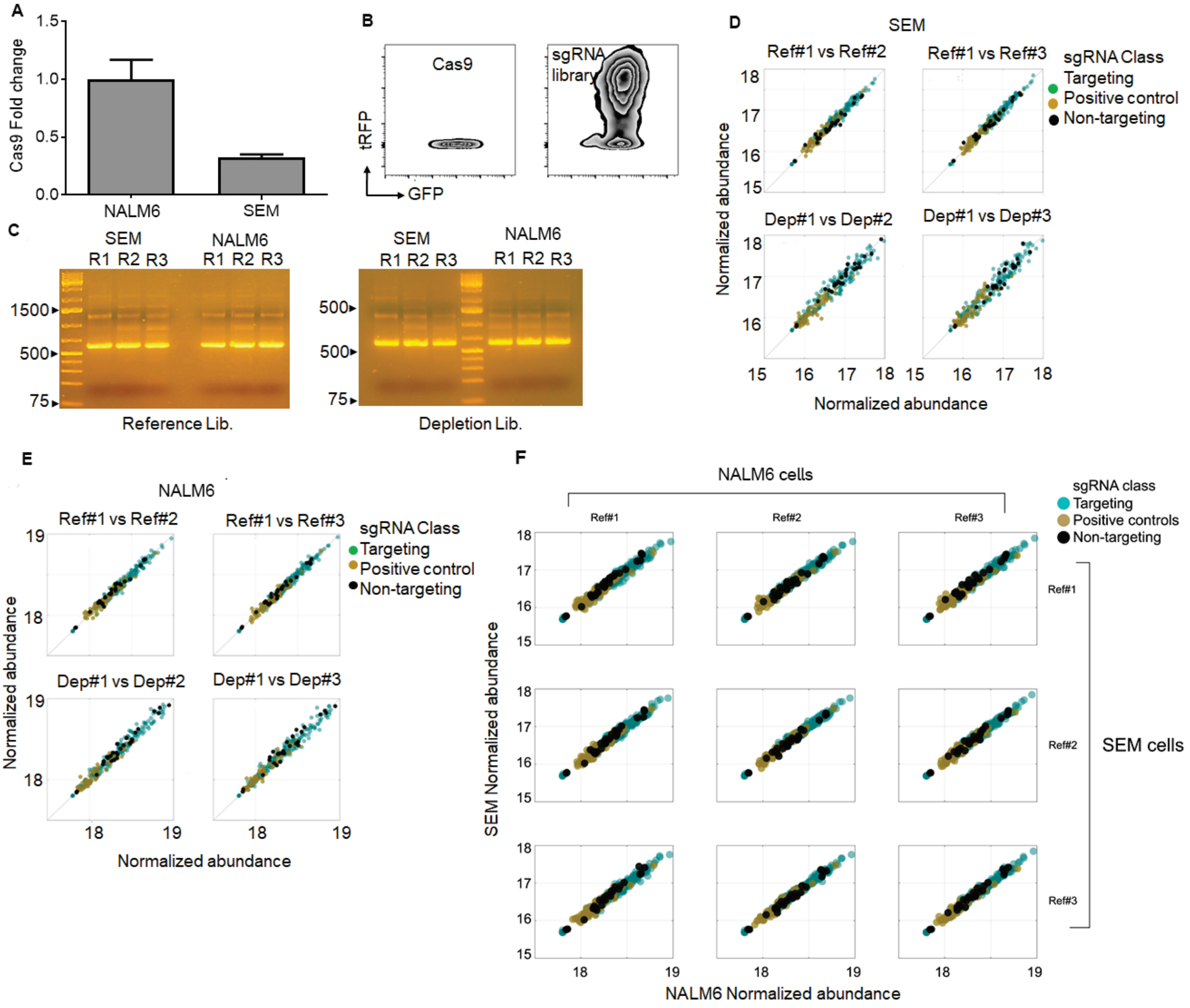

Figure S1. Sub-genomic CRISPR screen preparation. **A**. Cas9 expression in B-ALL cell line NALM6 and SEM prior to CRISPR screen. **B**. FACS plot showing transduction of NALM6 Cas9.EGFP with the undiluted lentiviral vector library, demonstrating effective production of virus with a high titer. **C**. Library preparation from the genomic DNA isolated from the sorted cells as Reference library and from expanded cells for 4 weeks as Depletion library. **D-E.** Intra-cell line Correlation plot of individual sgRNA variance-stabilized abundances in the reference and depletion libraries prepared in SEM and NALM6 cell lines between biological replicates. **F.** Inter-cell line correlation plots of individual sgRNA variance-stabilized abundances in the reference and depletion libraries prepared in SEM and NALM6 cell lines between biological replicates.

Figure S2

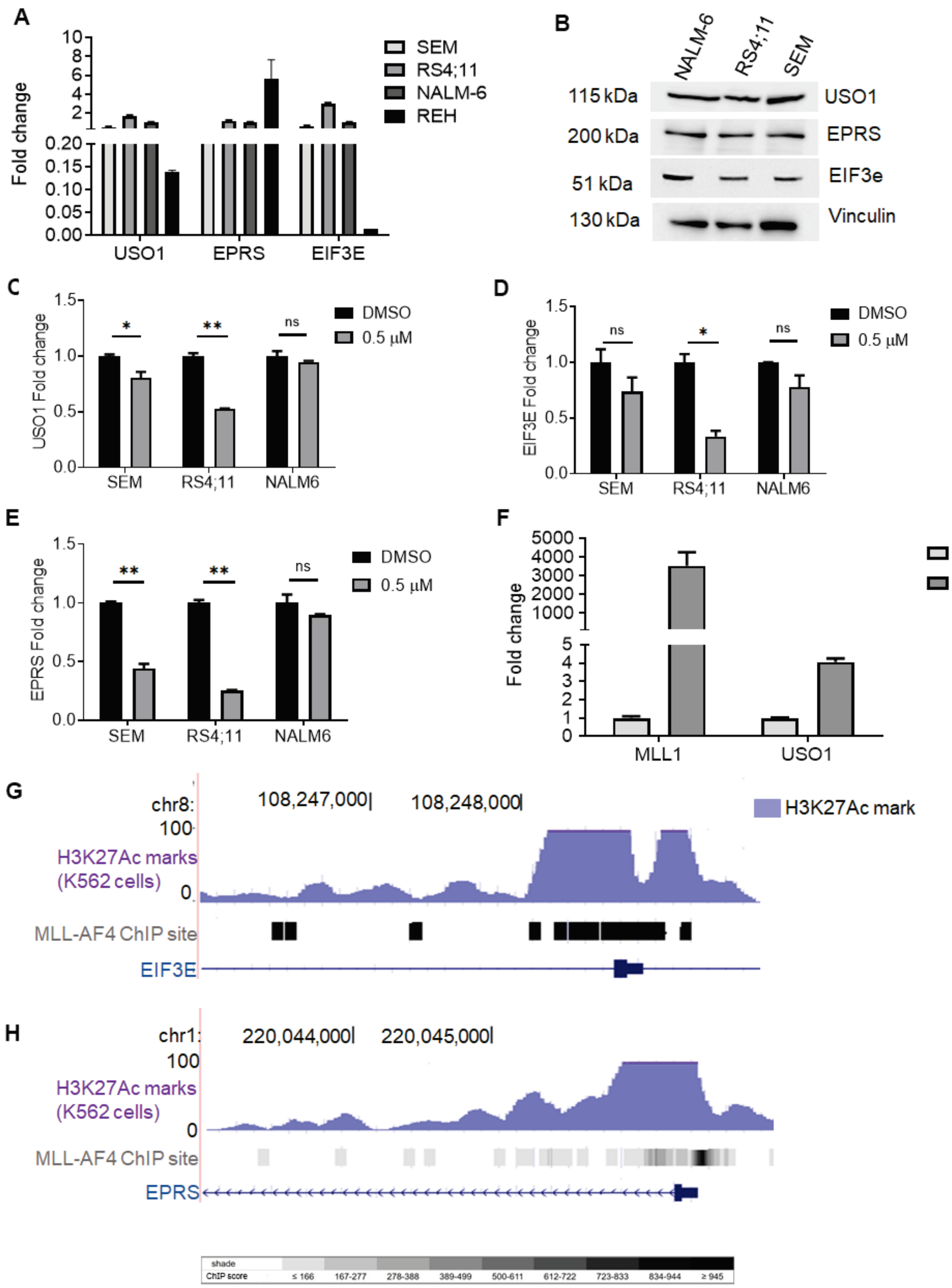

Figure S2. RBP genes expression in B-ALL cell lines. A. mRNA expression of RBP genes identified, measured by RT-qPCR (*USO1, EIF3E* and *EPRS*). Data was normalized to 18S expression level and is represented as fold-change from SEM cells, which are arbitrarily assigned a fold-change of 1. Cell lines examined include SEM, RS4;11 (carry the MLL-AF4 translocation), and NALM6 and REH (do not carry the MLL-AF4 translocation). **B**. Western blot to study basal level expression of *USO1, EIF3E* and *EPRS* in NALM6, SEM and RS4;11cells. **C-E.** RT-qPCR analysis of expression of USO1 (C), EIF3E (D), and EPRS (E) in SEM, RS4;11 and NALM6 cells treated with 0.5 µM EPZ5676. **F.** RT-qPCR analysis of USO1 in murine bone marrow cells with overexpression of MLL-Af4 (control versus MLL-AF4 bars). **G-H.** UCSC genome browser shots of the EIF3E and EPRS loci showing the *MLL-AF4* ChIP site(s) in the regulatory region as identified from the ChIP-Seq data from Lin *et. al.*(ref #21); *Courtesy: UCSC Genome Browser*). Shown are the H3K27Ac track in hematopoietic K562 cells (Blue), and MLL-AF4 binding sites represented as a grayscale score, with black indicating the highest score/highest number of reads from the dataset.

Figure S3

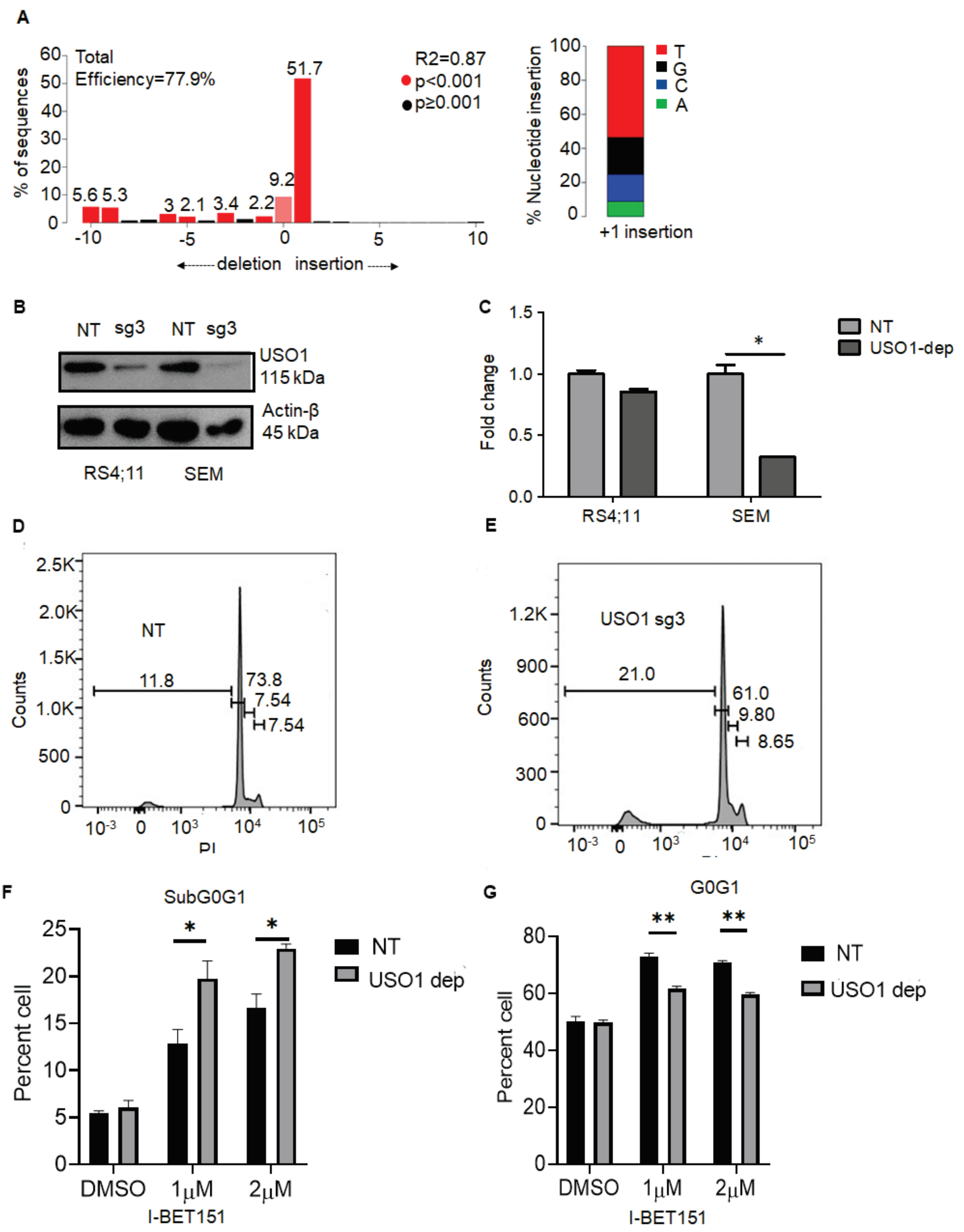

Figure S3. Targeting USO1 in B-ALL cells. A. Plot of insertions and deletions by position near the Cas9 cut site in SEM cells targeted with *USO1* sg3; as determined by Tracking of Indels by Decomposition (TIDE). Genomic sequence aberration is highest immediately adjacent to the cut site (designated as 0). **B.** USO1 depletion by transduction with sg3 in RS4;11 and SEM cells was confirmed by Western blot analysis. **C.** RT-qPCR of USO1 mRNA expression level, expressed as fold-change, in RS4;11 and SEM cells (t-test;$^*P < 0.05$). **D-G.** Cell cycle analysis using propidium iodide (PI) of control cells (NT) and USO1-depleted cells (D, E, respectively) following treatment with 1mM I-BET151. F-G. Quantitation of cells from cell cycle analysis in Sub-G0 (F) and G0/G1 (G) at 2 different concentrations of I-BET151(t-test; $^*P < 0.05$, $^{**}P < 0.001$).

Figure S4

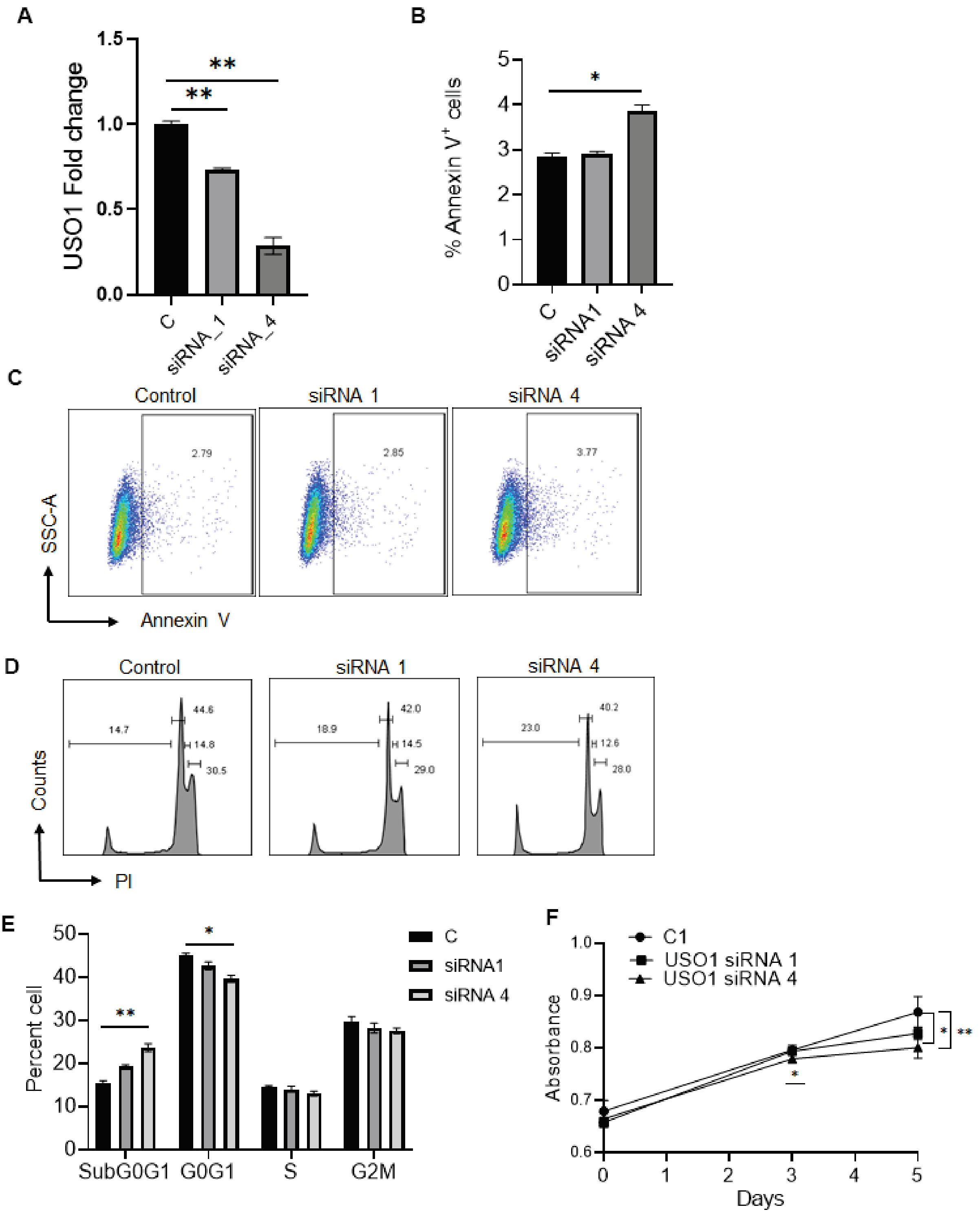

Figure S4. Modulation of USO1 expression by siRNA mediated knockdown. A. RT-qPCR analysis of USO1 expression levels, expressed as fold-change, in SEM cells that were subjected to nucleofection with a control siRNA or siRNA_1 or siRNA_4. **B-C.** Apoptosis induction, as measured by Annexin V staining, in SEM cells that were transduced with USO1-siRNAs. **D-E.** Cell cycle analysis, using propidium iodide staining, of USO1-siRNA-treated cells. **F.** MTS assays (Absorbance at 490nm) measuring the growth over time (days) of USO1-siRNA-treated SEM cells. (T-test; *P < 0.05, **P< 0.001).

Figure S5

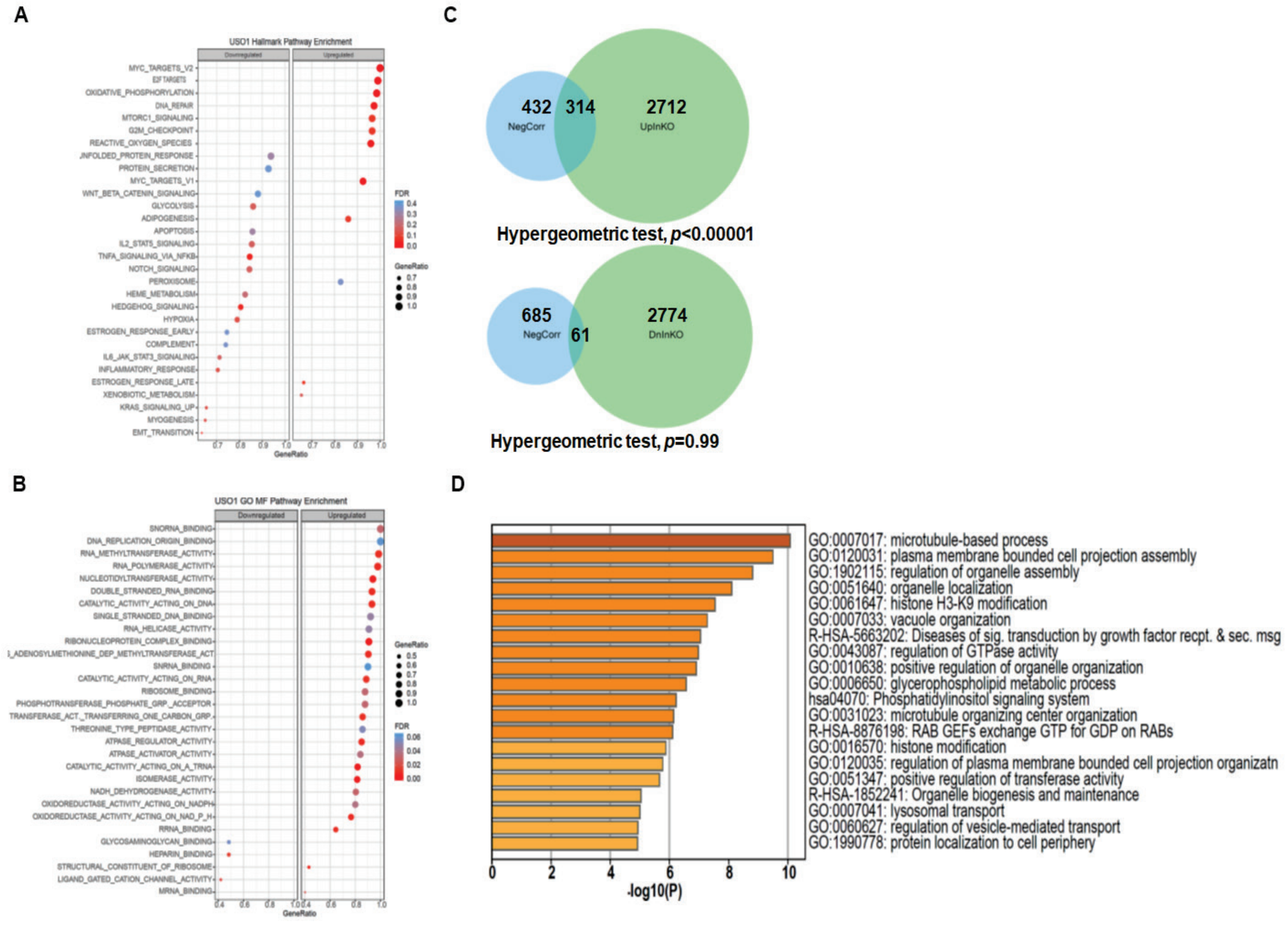

Figure S5. GO Molecular function analysis from RNA-Seq data. A-B. Differential expression analysis of *USO1* depleted SEM cells analyzed by GSEA with msigDB Hallmark gene sets and the GO Molecular function dataset, show enrichment of multiple RNA homeostasis related pathway. **C.** Venn diagrams showing overlap of *USO1*-negatively correlated genes in Target-Phase II ALL dataset with the genes that are significantly downregulated or upregulated in *USO1* depleted SEM cells. See Figure 4 for assumptions utilized in the Hypergeometric test. **D**. Enrichment analysis of the genes downregulated in *USO1* depleted cells that also have a high positive correlation with USO1 using Metascape. The data shows enrichment of several cancer-relevant pathways.

Figure S6

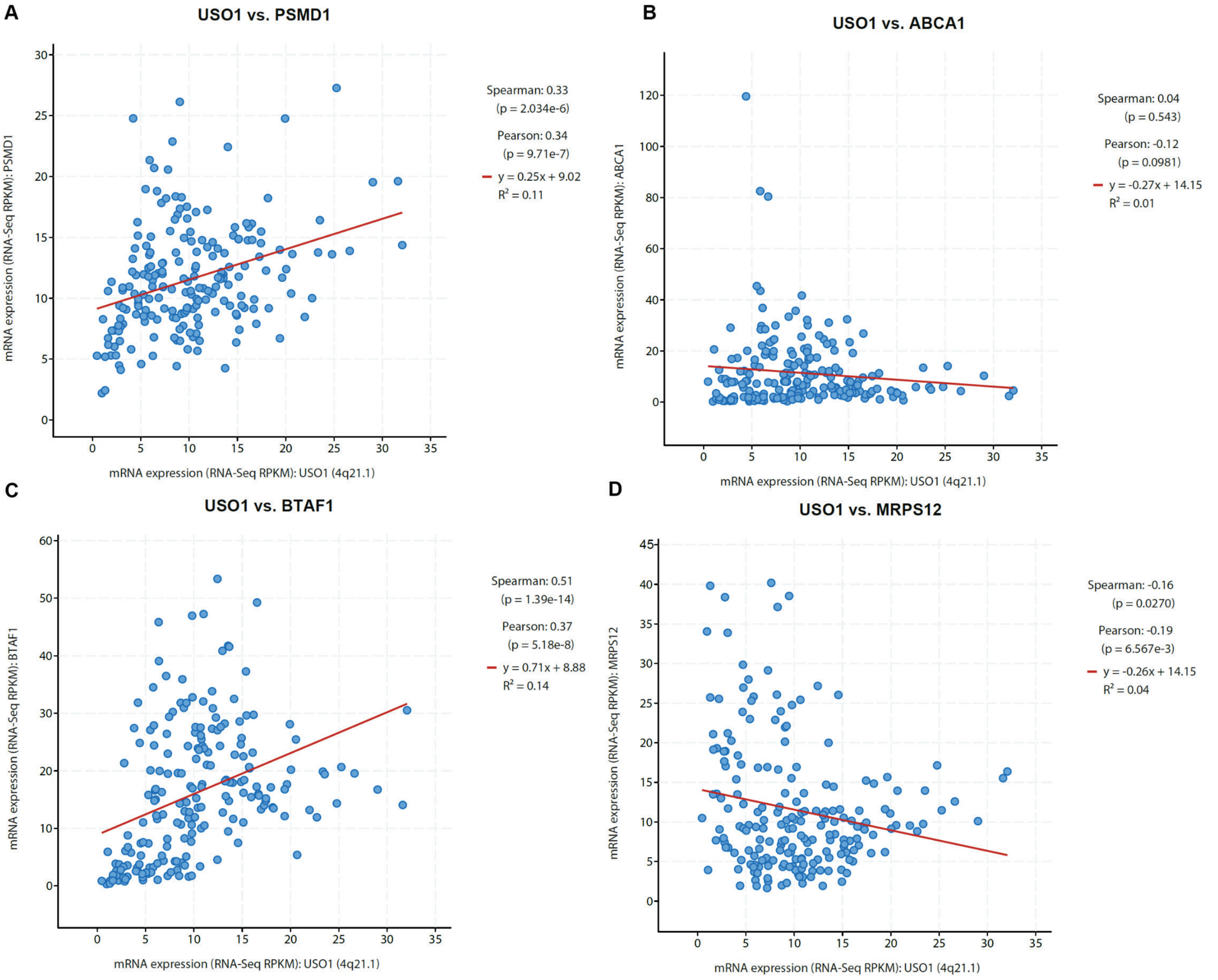

Figure S6. Correlation of USO1 with experimentally downregulated genes following USO1 knockdown. A-D. Genes downregulated in *USO1* depleted SEM, including *PSMD1*, *ABCA1, BTAF1 and MRPS12* show a strong positive correlation with USO1 expression in clinical B-ALL samples (*Courtesy: cBioPortal).*

Figure S7a

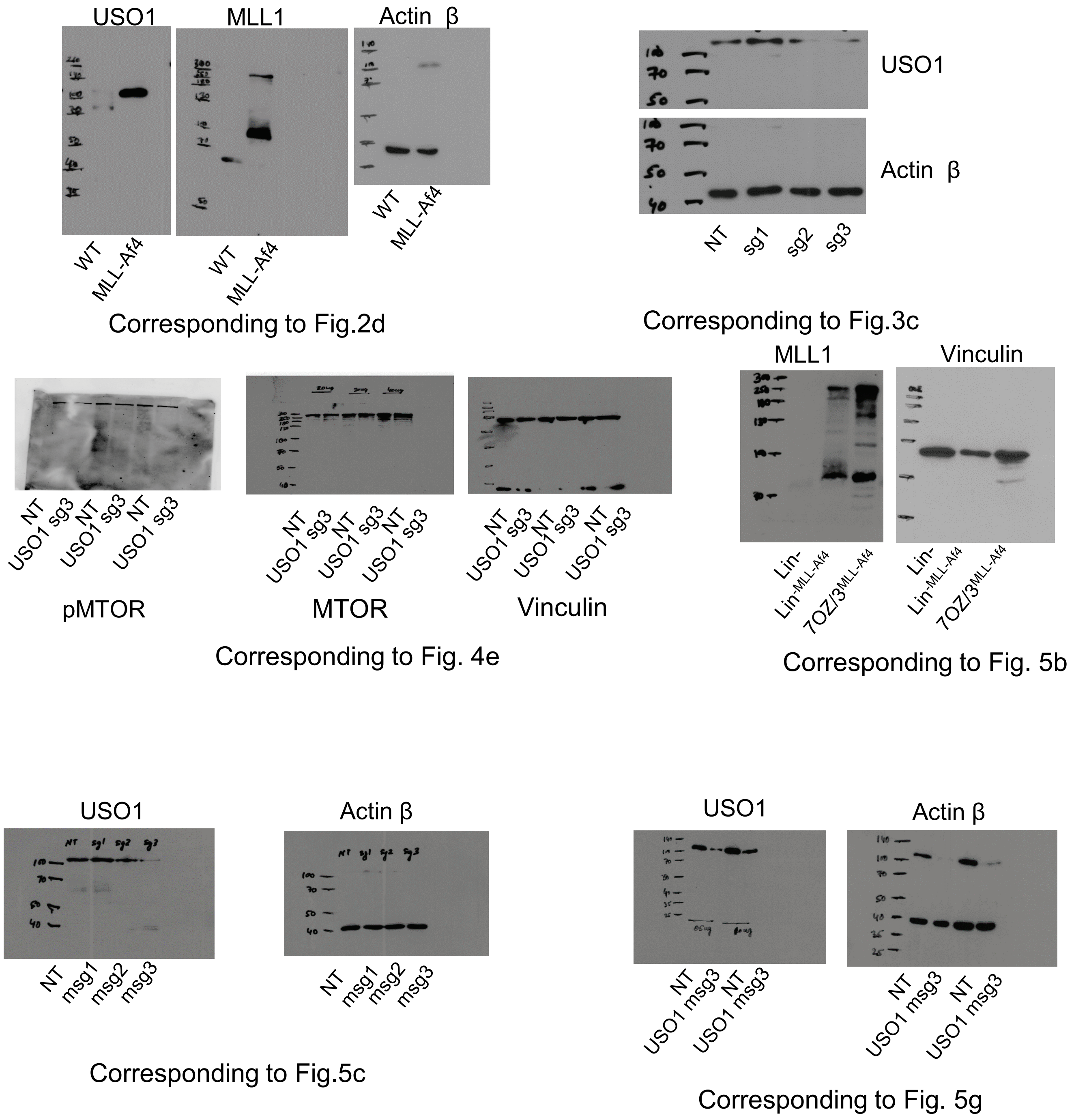

Figure S7a. Primary Western Blot Data. Each sub-panel lists the corresponding main panel in the main figures. These panels are the portion of the Western Blot scanned image that corresponds with the experiment being performed. In other cases, panels are showing the portion of a scanned image obtained by imaging on a ChemiDoc Imaging system from Biorad.

Figure S7b

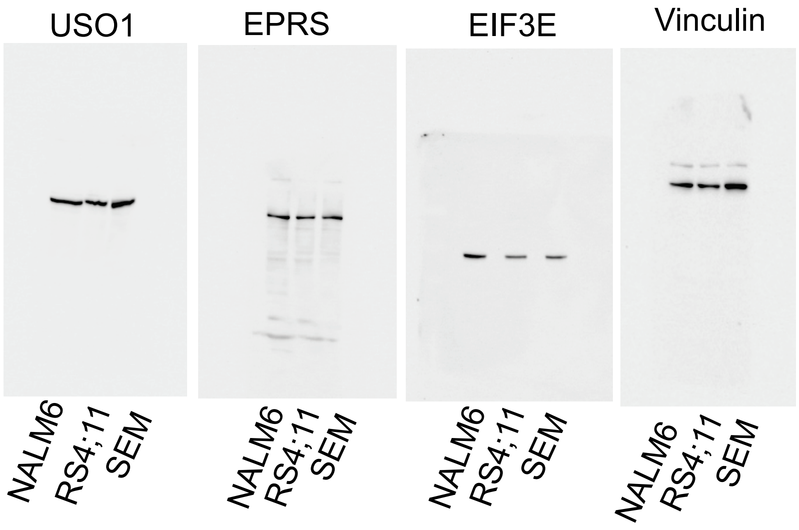

Corresponding to Fig. S2b

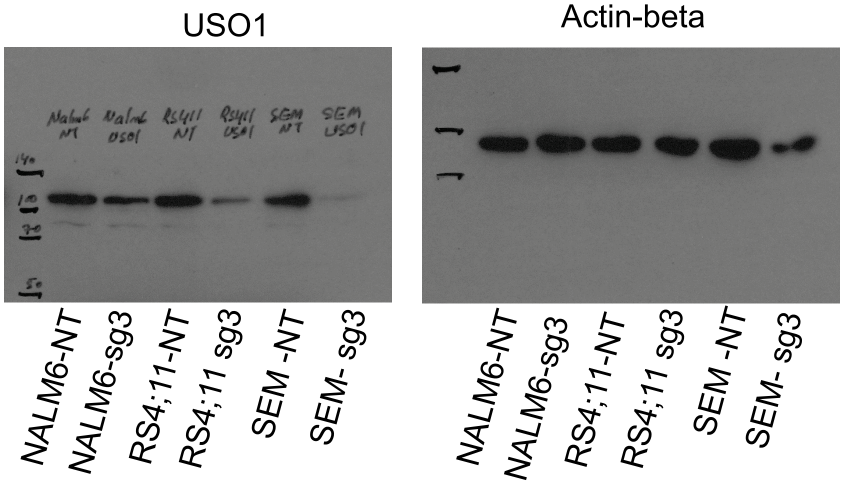

Corresponding to Fig. S3b

Figure S7b. Primary Western Blot Data. Each sub-panel lists the corresponding main panel in the main figures. These panels are the portion of the Western Blot scanned image that corresponds with the experiment being performed. In other cases, panels are showing the portion of a scanned image obtained by imaging on a ChemiDoc Imaging system from Biorad.

Gene Name	Guide RNA sequence
IGF2BP3	AGCTTGGTCCTTACTGGAAT
IGF2BP3	AAGCACCAGATGCTAAAGTG
IGF2BP3	C CAC CGT AAA GAA AAT GCG G
IGF2BP3	A GGC GCA GAG GCA AAT CAC A
IGF2BP3	G CTG GGC GGC CAT TTC ATC A
BAZ1A	C AGT GCA TAA GCC AAA TCG G
BAZ1A	T GGT GTC AAA TCA TAC ACT G
BAZ1A	T CCA GTA TCG TCT ATA CAT G
BAZ1A	G TAA GCG CGA ACG ATG GGT A
BAZ1A	T TTC CTG ATG GAG TAA CCC T
USO1	A GGG TGA TAA GAT CGA CAG A
USO1	G GAC TTA CTA GCG GAT TCC A
USO1	A GCT ACT GGT AGC ACC AGG A
USO1	T CAG CTG GCC AGT TAT TAT G
DDX3Y	TATGAACACCACTACAAGGG
DDX3Y	C AGA TTC GGG ACT TAG AAC G
DDX3Y	C AGT TTA GCG ATA TTG ACA T
DDX3Y	A TGA TAA AGA CAG TTC AGG T
DDX3Y	G TAT ATA TCT GAC TCA GTA T
MBNL1	G TAT GTC GAG AGT ACC AAC G
MBNL1	CAACGTGGCAATTGCAACCG
MBNL1	C TTG GTG CAA CTG AAA ACA T
MBNL1	G TAA CCA ACA TTG GTG CAG T
MBNL1	T GCC CAA TAC CAG GTC AAC C
TUBB6	G CTG ACA ACG CCC ACC TAC G
TUBB6	C AGC TGG TGC ACC GAC AGT G
TUBB6	C CAT GGC CGC TAC CTG ACC G
TUBB6	C TGG CCC GGG AAG CGC AGC G
TUBB6	GTTGCAGGCCAGACGGGTGC
DDX21	C CTA CAT TCA TCG ATC CGG G
DDX21	G CAA CAT TAA ATA CCC AAT G
DDX21	G ATG TCA TCC GAG TAT ATA G
DDX21	C ATC ACA AAA AAG CTG TCA G
DDX21	CTAGTTTGCCATTCTGTATG
LUC7L3	G TAG AAC GTA GGA TCA GAC G
LUC7L3	C ATC AAA TGG TCA TCT ACC C
LUC7L3	G TAT TTG TGA ACA ATT CCG C
LUC7L3	T TTA GAC GCT CAT CAC GAT C
LUC7L3	A ACT AAT TTC ATC ATC CCC T
LARP7	T GGA AGG CAC CAG AAT CCG G

**LARP7**	A TCA CTC CAC TCA CGA ACT G
**LARP7**	A GAC TAT CAG GAT CCG GAG A
**LARP7**	T GCT AGA TTT AAA ACT CCT G
**LARP7**	GAGAAGTTCAGCTGTTGTAG
**IQGAP1**	C TAC GCG TCC AAC CAG CGA G
**IQGAP1**	ATCAGCGGAGGTACCGACAG
**IQGAP1**	C CTG TCG AAC TAA GTA TCC A
**IQGAP1**	G AAT GAT ATC CAA GCT TGC G
**IQGAP1**	CCGTGGATACTTAGTTCGAC
**CSTF3**	C TCC TTC CTC ACC TGC TCC G
**CSTF3**	G CTG AGG CCA TGT CAG GAG A
**CSTF3**	C CCA GAG AAG GTG AAG AAA G
**CSTF3**	A GCT GAG TAT GTC CCA GAG A
**CSTF3**	GGTTTACACCCTGTACCTGG
**EPRS**	A ATG TAT ATC AAA ACA CAC G
**EPRS**	C ATG TTC ACG ACT GAA CGT G
**EPRS**	C TCC AGT TCA TGT AAA ACG T
**EPRS**	A GAA TGC TTA CTG TCC CTG A
**EPRS**	A GTA GAG TAT AAG CCT GTG T
**ANXA1**	GGTGACGCAGAACTCCCCTG
**ANXA1**	T CAC ACC AAA GTC CTC AGA T
**ANXA1**	AGACCTTCGACGTGAATCGC
**ANXA1**	C CTT ACA GGT CAC CTT GAG G
**ANXA1**	A AAA TCT CCA GAT GTG TCT G
**EIF3E**	T ACA GCA GTC ATA ACA AAC A
**EIF3E**	T AAT ATT ATC GCG ACC TTT G
**EIF3E**	CTT CTT AGT GAT ACC AAC A
**EIF3E**	A ATG CTT TAA GTT CAC TCT G
**EIF3E**	C AAA ATT CCA GTA CGA ATG T
**ANXAP21**	C AGG GAC TGC TAG TCA CAA G
**ANXAP21**	GGATCAGCGTCATGACCGAG
**ANXAP21**	A AGG AAC TGA AGT TCC CAA G
**ANXAP21**	T GAC TGA CCC AAA TGC ACT T
**ANXAP21**	G TTA GTC AAA ATG TTG ACA G
**CAMK2B**	C ACC GTC ATC CAT AAC CCA G
**CAMK2B**	C TCA CCT GAG AAA TTC CGT G
**CAMK2B**	G GCG AGA GAG TAC TAC AGC G
**CAMK2B**	G TGC ATC ATG GAT GCT ACC G
**CAMK2B**	C TCT TTG CGA AGG ACC TCA G
**PAICS**	A TTG GAA TCA TTT CAC ACT G
**PAICS**	T ACG AAT TGT TAG ACA GTC C

**PAICS**	G CTA GAA AAA ACC ACC TGG A
**PAICS**	A TGC CAA TAA TGA CCC ACA G
**PAICS**	C TTA CGA TTT GAC TTA TAT G
**FLNA**	C GTC AAA GCA GGG AAC CAC G
**FLNA**	A GTG GAG TAC ACG CCT TAC G
**FLNA**	T GGT GAC GAT ATA GGT TCC A
**FLNA**	A GTC GAT GTC AGC TTC GGC G
**FLNA**	T TAT CCA CGT ACA CCT CGA A
**SLTM**	A AAC CTC TTT GCT TGA CCA G
**SLTM**	A ACT ATG TCT TCA AGC ACA G
**SLTM**	G CAT TAG AAT AAT TCG TGA A
**SLTM**	T CTA ATG TAG GCT ATT GAA G
**AHNAK**	G GGC CCA AGT ATG AAC ATT G
**AHNAK**	G TGA TGC TAC CCC CAA TTT G
**AHNAK**	G GCT CTT CAA GTG TAG ATC G
**PPIG**	T CTG GAC GGA CAG TAG ACT G
**PPIG**	G TGG TGA AAG ATG GAT CAA G
**PPIG**	A GCT AGC AAA CCG TTT GCG G
**PPIG**	G GAG GGG AAT CTA TCT ATG G
**PPIG**	A AAG GGC CAA ATC TAA AAG T
**BAG2**	T CAA CGC TAA AGC CAA CGA G
**BAG2**	T CCA AAA TAG CCA GGA CAT G
**BAG2**	C ACC TCA TCA ATA ATC CTT G
**BAG2**	G AGC GAC ATT AAA TGA CTC T
**BAG2**	GATCAACGCTAAAGCCAACG
**MSH2**	C TGT CTC TGG CCA TCA ACT G
**MSH2**	C ATG GAT AAG AAC AGA ATA G
**MSH2**	A GGG GGT GAT CAA GTA CAT G
**MSH2**	A CAT CAT TGA GTG TCT GCA T
**LMNA**	GCGCCGTCATGAGACCCGAC
**LMNA**	C CGT CAT GAG ACC CGA CTG G
**LMNA**	A GTT GAT GAG AGC CGT ACG C
**LMNA**	G GAC AGA GAC TGC TCG GAG T
**ANAPC4**	C CTG CAG CAT CTA GTC CAA G
**ANAPC4**	G TCA CAG AAG TCT CTA CCA A
**ANAPC4**	CTCAAAATGCATGGTACGGG
**ANAPC4**	AAAGACACTGTAGGACGTGA
**RAD50**	C TAG GAA CGT GAG TTA AGC A
**RAD50**	A AGC GGC GTG ATG AAA TGC T
**RAD50**	A AAC AGC ACA AGT TAG ACA C
**RAD50**	A AAA ACT GCC AAC CAA CTG A

**RAD50**	T ACA TTT GTA CAC GAT CCC A
**VIM**	A CAG CAT GTC CAA ATC GAT G
**VIM**	TCCTACCGCAGGATGTTCGG
**VIM**	A GAA GAA TGG TAC AAA TCC A
**VIM**	C AAC GAC AAA GCC CGC GTC G
**VIM**	C TCT ACG CCT CGT CCC CGG G
**S100A10**	G GAT AAA GGC TAC TTA ACA A
**S100A10**	A TCT CAA ATG GAA CAC GCC A
**S100A10**	G GAG GAC CTG AGA GTA CTC A
**S100A10**	C ACT TAC TTC CAA AAA TCC A
**S100A10**	G GAC AAA ATA ATG AAG GAC C
**PALLD**	A CGA AAA CTT CGC TTC AAG G
**PALLD**	G CTG CTG AGA GGG AAA CGA A
**PALLD**	G AGG CCT TTG AGG ACG ACA C
**PALLD**	G GAA CTG CAA AAC ACA GCC G
**PALLD**	A CCT GAG GTG ACA ACC AGC T
**SKIV2L2**	T CTG TGT AAA TAA CAT GAC A
**SKIV2L2**	T GTT GAA GGG TGT ACA CAT G
**SKIV2L2**	T TGT CTT GTT ATG ACC ACA G
**SKIV2L2**	T TTC CAT TTG ACC TAC AAC A
**SKIV2L2**	A CCA AAT TAG ATT TCA ACA C
**SMARCAD1**	A TGA CCG TAG TCT GTT TCG A
**SMARCAD1**	T GTC GGT AGT TCA CTA GAT G
**SMARCAD1**	C TGG CAT ACC TCT ATC AGG A
**SMARCAD1**	T GCA TCA CCA AAC ATC AGC A
**SMARCAD1**	A CCT CCA TTT CCA GTA AGC A
**OGT**	T GGC AAT AGA CAC CTA CAG G
**OGT**	G CCA ATA TCA AAC GAG AAC A
**OGT**	T GGC ACA AAC TTC CGA GTG A
**OGT**	G GCA CGC GTA TAA CAC TGC A
**OGT**	C TAT ACA CTT CCA GTG TCG A
**C1QBP**	G GCC CGC TAA ACC TCA CCG T
**C1QBP**	A TTA GTG CGG AAA GTT GCC G
**C1QBP**	G CGT GCG CGC AGG TTC CGA G
**C1QBP**	G ATA ATG ACA GTC CAA CAC A
**C1QBP**	GGACAGAAGCGAAATTAGTG
**HSPD1P6**	G AAA GCT TGA AGT TTG ATC G
**HSPD1P6**	G GTC TTA TCT AAA GAC AGT G
**HSPD1P6**	T AGC TAT GCC AAA GGT TGG T
**HSPD1P6**	C ACC CCT GAA GAA ATT GCG C
**HSPD1P6**	A TAA TCA CTG AAG ATG TTG G

**PSDM14**	A AAC ACA TCA ATC ACT CTG A
**PSDM14**	A AAG CCA ACA ACC AAA GCC A
**PSDM14**	A TCA AAC CCA TAA CTT CCA T
**PSDM14**	A GAC AAA CAA CTT CGA ATC T
**PSDM14**	A GCC TTG TCG GAG AGA GCT G
**MYC**	CTTCGGGGAGACAACGACGG
**MYC**	AACGTTGAGGGGCATCGTCG
**MYC**	GCCGTATTTCTACTGCGACG
**MYC**	CGAGGAGAGCAGAGAATCCG
**MYC**	TGCGTAGTTGTGCTGATGTG
**HOXA7**	CTCACAGAGAAGCGGCTACG
**HOXA7**	GCCTGCGACAAGACGGACGA
**HOXA7**	CAAAGGGGCTCTGATAAAGG
**HOXA7**	TACCTGAAGACCGCATCCAG
**HOXA7**	GGAGCAAAGGAGCAAGAAGT
**MEIS1**	GGTGGCCACACGTCACACAG
**MEIS1**	ATGCGGGTCCCCATACATCG
**MEIS1**	CACAGCTCATACCAACGCCA
**MEIS1**	ATCATGATCTCTGTTCCAAG
**MEIS1**	CTTTAAAGCGTCATTGACAG
**CDK6**	GCCCGCGACTTGAAGAACGG
**CDK6**	AACACTCCAGAGATCCACGG
**CDK6**	GGAAACTATAGATGCGGGCA
**CDK6**	TGGCTCACCTGACCACGTTG
**CDK6**	CCAGCAGTACGAATGCGTGG
**HOXA9**	CAAAGTGTGAGTGTCAAGCG
**HOXA9**	GCGTTGGCCGCTATGCGCCG
**HOXA9**	CGACGGTGTTTGGCGCCTCG
**HOXA9**	ATGGTGGTGGTACACCGCAG
**HOXA9**	CAGGTTTAATGCCATAAGGC
**CoA5**	CATAGGAAGGAAAATCACCT
**CoA5**	TATTATGAGGACAAGCCGCA
**CoA5**	CGTCATGCCTAAGTATTATG
**CoA5**	TCTGCTGCAGTCGGACTGTG
**CoA5**	CGGCGCGTGCGCGGGCCTGA
**RPL11**	TGTCCACTGCACAGTTCGAG
**RPL11**	GGATGCGAAGTTCCCGCATG
**RPL11**	CTCACCTTTGGAAAACACAG
**RPL11**	AACTCATACTCCCGCACCTG
**RPL11**	TTCATTTCTCCGGATGCCAA
**SRSF2**	AACGACTCCGACTCCGGGAT

**SRSF2**	TCGAGCGGCTGTAGCGAGAT
**SRSF2**	CTTGTCGTGAAAGCGAACGA
**SRSF2**	GGAGGGTATGACCTCCCTCA
**SRSF2**	CGGGTGCAAATGGCGCGCTA
**RPS27**	TTGTGCATGGCTAAAGACCG
**RPS27**	CTGTCCCTCTTAGCTCGCAA
**RPS27**	TTCCTCCTGAAGGAACATCC
**RPS27**	TTCCTCCAGGATGTTCCTTC
**RPS27**	CTCCAGGATGTTCCTTCAGG
**HEATR1**	ATTGAACTGATGTTAGACAG
**HEATR1**	ACAACAAAGGAACTTTACGC
**HEATR1**	CAGGAACATTACATAAGTGA
**HEATR1**	ATACAACATCTAATGTTCTG
**HEATR1**	GCCCCGTAAAAGAAGTTCGT
**HIST1H2AH**	GCTCGGCATAATTACCCTTG
**HIST1H2AH**	GAAGCCCAGCCCGAGAAGAG
**HIST1H2AH**	TCTGGACGTGGCAAGCAAGG
**HIST1H2AH**	GGCTGGGCTTCAGTTCCCCG
**HIST1H2AH**	GAGGTGACGCGGGATGATAC
**Atr(LOC648152)**	TGACGTGCGAAAACAAGATG
**Atr(LOC648152)**	CTGTGTGAGATGGTCAAGCA
**Atr(LOC648152)**	AGTCAATTAGATGAACACAT
**Atr(LOC648152)**	AGAACCTATTATCTCACAGT
**Atr(LOC648152)**	TAATTATAGAATTAAACATG
**STAT5A_1**	GCACTACGCCACGCAGCTCC
**STAT5A_2**	TACACCCACCTGGAGCTGCG
**STAT5A_3**	ACCAGGACTGTAGCACGTCC
**STAT5A_4**	CATTGACTTGGACAATCCCC
**STAT5A_5**	CGTGCACATGAATCCCCCCC
**BCL-2**	GGCCTTCTTTGAGTTCGGTG
**BCL-2**	TGGACATCTCGGCGAAGTCG
**BCL-2**	TGTCGCAGAGGGGCTACGAG
**BCL-2**	CTGACGCCCTTCACCGCGCG
**BCL-2**	GCCCCATCCAGCCGCATCCC
**NT1**	ACGGAGGCTAAGCGTCGCAA
**NT2**	CGCTTCCGCGGCCCGTTCAA
**NT3**	ATCGTTTCCGCTTAACGGCG
**NT4**	GTAGGCGCGCCGCTCTCTAC
**NT5**	CCATATCGGGGCGAGACATG
**NT6**	TACTAACGCCGCTCCTACAG

**NT7**	TCATCTTACATCTGGGAGAC
**NT8**	GGGCCCGCATAGGATATCGC
**NT9**	TAGACAACCGCGGAGAATGC
**NT10**	ACGGGCGGCTATCGCTGACT
**NT11**	CGCGGAAATTTTACCGACGA
**NT12**	CTTACAATCGTCGGTCCAAT
**NT13**	GCGTGCGTCCCGGGTTACCC
**NT14**	CGGAGTAACAAGCGGACGGA
**NT15**	CGAGTGTTATACGCACCGTT
**NT16**	CGACTAACCGGAAACTTTTT
**NT17**	CAAGTTTTCTGAAAGGCAAT
**NT18**	AAAGATATAGCAAATTATGG
**NT19**	TTCACGTCGTCTCGCGACCA
**NT20**	GTGTCGGATTCCGCCGCTTA
**NT21**	GTTAGGAATAAAAGCTTTGA
**NT22**	ACTTCCCACTTCTTAGGTTG
**NT23**	CCATTCACAATCCCACTACA
**NT24**	TGTTTTGCATGTTGCATAGG
**NT25**	TAAGCCTCATGAAGGAGGGG
**NT26**	GGTACTGGAAGTCCGAAACC
**NT27**	AAATAATATGCATCTCTCGA
**NT28**	AGCTGGACTCTGTAGAAATC

Table S1. Short guide RNA (sgRNA) sequences.

Genes	Primer Sequence
USO1-Forward Primer	TACCGCTTGGAAGTGGGTAT
USO1-Reverse Primer	AAATCTTCACTCTGTCTTGTGGAAT
EPRS- Forward Primer	GGG AAG GCT TAT GTG GAT GA
EPRS- Reverse Primer	CTC GCA AAC AAC AGG ACT
EIF3E-Forward Primer	CCCTCACCTCCTTTACAGAG
EIF3E-Reverse Primer	GCACAGACTCCCTTTTCTTT
MRSP12-Forward Primer	CCTCTTCCATCAGGACCACT
MRSP12-Reverse Primer	CAGTGTCTTGTCCCAGCAG
TRFC-Forward Primer	TGGACAGCACAGACTTCACC
TRFC-Reverse Primer	CCAGGATTCTCCACCAGG
NOP16-Forward Primer	AGGGTGTAAGGCAAGGAGGT
NOP16-Reverse Primer	ATCACCCACCCTGAAAACA
DDX21-Forward Primer	GCAGCAGTTATTGGGGATGT
DDX21-Reverse Primer	TGAATTCTGGGACAGCTCCT
PSMD1-Forward Primer	CTGTGGTTGGCGTCCTTGTAT
PSMD1-Reverse Primer	CAAATGTGGATGGTTTACAG
Human 18s Forward Primer	GTAACCCGTTGAACCCCATT
Human 18s Reverse Primer	CCATCCAATCGGTAGTAGCG
Uso1-Forward Primer	CCAGGGAAGCAAAATACAGA
Uso1-Reverse Primer	GCACATAAGCCTTGGACC
L-32 Forward Primer	AAG CGA AAC TGG CGG AAA C
L-32 Reverse Primer	TAA CCG ATG TTG GGC ATC AG

Genes	ChIP primer sequence
USO1-Fw	TGGCTGAACGGCAAGATGAAT
USO1- Rev	CTGGGATTTCAGGAGCGGA

Genes	sgRNA sequence
Ms-NT	GCGAGGTATTCGGCTCCGCG
Uso1-1	GGTAACGTAGAGGAAGCTACG
Uso1-2	GTCGGTCATCCAGTAACGTAG
Uso1-3	GACTGCTGGACATTATTACAG

CRISPR Lib. primers	Sequence
Part I Illumina Universal Adaptor with variable region and vector seq.	ACACTCTTTCCCTACACGACGCTCTTCCGATCTT AA TGGAAAGGACGAAACACCGA
PartII Illumina Index adaptor	GACTGGAGTTCAGACGTGTGCTCTTCCGATCTT TTAGTTTGTATGTCTGTTG
Rest Illumina universal adaptor	AATGATACGGCGACCACCGAGATCTACACTCTT TCCCTACACGACGC
Rest Illumina Index adaptor	CAAGCAGAAGACGGCATACGAGATCGTGATGT GACTGGAGTTCAGACGTGTGC

Table S2. Primer sequences. Please see text for details.
